# Understanding the burden of antibiotic resistance: a decade of carbapenem-resistant Gram-negative bacterial infections in Italian intensive care units

**DOI:** 10.3389/fmicb.2024.1405390

**Published:** 2024-06-05

**Authors:** Giovanni Scaglione, Matilde Perego, Marta Colaneri, Camilla Genovese, Fabio Brivio, Alice Covizzi, Bruno Viaggi, Alessandra Bandera, Andrea Gori, Stefano Finazzi, Emanuele Palomba

**Affiliations:** ^1^Department of Infectious Diseases, Luigi Sacco Hospital, Milan, Italy; ^2^Department of Biomedical and Clinical Sciences “L. Sacco”, University of Milan, Milan, Italy; ^3^Laboratory of Clinical Data Science, Department of Public Health, Mario Negri Institute for Pharmacological Research IRCCS, Ranica, Italy; ^4^Centre for Multidisciplinary Research in Health Science (MACH), University of Milan, Milan, Italy; ^5^Department of Anaesthesiology, Neuro-Intensive Care Unit, Careggi University Hospital, Florence, Italy; ^6^Department of Pathophysiology and Transplantation, University of Milano, Milan, Italy; ^7^Infectious Diseases Unit, IRCCS Ca’ Granda Ospedale Maggiore Policlinico Foundation, Milan, Italy

**Keywords:** epidemiology, multidrug-resistant, intensive care unit, gram-negative, carbapenem-resistant, hospital-acquired infections

## Abstract

**Introduction:**

In patients admitted to intensive care units (ICUs), Gram-negative bacteria (GNB) infections pose significant challenges due to their contribution to morbidity, mortality, and healthcare costs. During the SARS-CoV-2 pandemic, Italy witnessed a rise in healthcare-associated infections (HAIs), with GNBs involved in a substantial proportion of cases. Concerningly, carbapenem-resistant GNBs (CR-GNBs) have increased worldwide, posing therapeutic challenges.

**Methods:**

Retrospective multicentre study analysing data from over 299,000 patients admitted to Italian ICUs from 2013 to 2022.

**Results:**

The study revealed an average of 1.5 infections per patient, with HAIs peaking during the pandemic years. Ventilator associated pneumonia (VAP) emerged as the most common HAI, with *Klebsiella* spp. and *Pseudomonas aeruginosa* predominating. Alarmingly, CR-GNBs accounted for a significant proportion of infections, particularly in VAP, bloodstream infections, and intra-abdominal infections.

**Discussion:**

Our findings underscore the pressing need for enhanced infection control measures, particularly in the ICU setting, to mitigate the rising prevalence of CR-GNBs and their impact on patient outcomes. The study provides valuable insights into the epidemiology of HAIs in Italian ICUs and highlights the challenges posed by CR-GNBs, especially in the context of the SARS-CoV-2 pandemic, which exacerbated the issue and may serve as a crucial example for the management of future viral pandemics.

## 1 Introduction

Infections caused by gram-negative bacteria (GNB) are significant contributors to morbidity, mortality, and healthcare costs among patients admitted to intensive care units (ICUs) (Saha and Sarkar, 2021). ICU patients face heightened vulnerability to GNB infections due to frequent invasive medical procedures and compromised immune responses resulting from trauma, surgery, and underlying medical conditions (Bassetti et al., 2019). Hence, recent data from the European Centre for Disease Prevention and Control (ECDC) indicate that of all patients staying in an ICU for more than two days, 4% presented with ventilator-associated pneumonia (VAP), 3% with bloodstream infection (BSI), and 2% with urinary tract infection (UTI); in almost all cases these conditions were associated with the presence of an invasive device (European Centre for Disease Prevention and Control, 2023).

In Italy, the incidence of healthcare-associated infections (HAIs) increased during the SARS-CoV-2 pandemic from 15.4% in 2006–2007 up to 24.5% in 2020–2021 (Barchitta et al., 2023), with GNBs involved in more than three-quarters of cases (Temperoni et al., 2021; Scaravilli et al., 2022). The increased rates of carbapenem-resistant GNBs (CR-GNBs) worldwide, which might have been exacerbated during the SARS-CoV-2 pandemic, poses a serious threat, leading to limited treatment options, prolonged hospital stays, and increased mortality rates (Daoud and Dropa, 2023).

Pathogens listed by the World Health Organization (WHO) as Priority 1 (World Health Organization [WHO], 2017), including carbapenem-resistant *Pseudomonas aeruginosa* (CRPA), *Klebsiella pneumoniae*, and *Acinetobacter baumannii*, are of particular concern and have increased over the last decade (van Duin and Doi, 2017; Kinross et al., 2022; Torrens et al., 2022). Italy was placed among the worst-performing nations in Europe, characterized by alarmingly high levels of antimicrobial resistance (AMR), with hyper-endemic levels of these microorganisms (European Centre for Disease Prevention and Control, 2017).

However, studies regarding the burden of CR-GNBs in Italian ICUs, which faced great pressure during the COVID-19 era, are scarce and conflicting for both the pre-pandemic (until 2019) and pandemic (2020–2022) periods (European Centre for Disease Prevention and Control, 2017; Pace et al., 2023), leading to possibly even greater uncertainty in the decisions on appropriate antibiotic empiric regimens in this scenario. Understanding the rates and trends of CR-GNB infections over time is crucial for informing clinical decision-making and improving patient outcomes in the face of rising AMR. Therefore, this study aimed to investigate the rates and trends of CR-GNB infections in Italian ICUs over the past decade. Specifically, we focused on WHO priority pathogens *Pseudomonas aeruginosa*, *Klebsiella* spp., and *Acinetobacter* spp., examining their distribution across different infection sites, including VAP, BSI, intrabdominal infection (IAI), and UTI. By assessing differences before and after the SARS-CoV-2 pandemic, we aim to provide valuable insights into the impact of this global health crisis on the prevalence of multidrug-resistant microorganisms in critically ill patients.

## 2 Materials and methods

### 2.1 Study design

We conducted a multicentre retrospective and observational registry-based analysis as part of the PROSAFE (Promoting Patient Safety and Quality Improvement in Critical Care) research project. The PROSAFE study was conceived as a prospective observational project aimed at electronically collecting data on patients admitted in over 210 Italian ICUs using a software developed by the GiViTI (Gruppo italiano per la Valutazione interventi in Terapia Intensiva) (Finazzi et al., 2020).

### 2.2 Data collection

Data collection is ongoing since 2011 through an electronic case report form (eCRF) and is performed by senior ICU specialized physicians working in the participating centers. Our study focused on data collected from 1 January 2013 to 31 December 2022. Two time periods were identified, the “pre-SARS-CoV-2 pandemic” period, between 2013 and 2019, and the “SARS-CoV-2 pandemic” period, from 2020 to 2022.

All patients admitted to general Italian ICUs participating in the PROSAFE project were included in our study.

To ensure data integrity, cross variable checks were performed during data collection and inconsistent or missing data were reported in the eCRF. Validity, according to GiViTI metrics, corresponded to the data regarding patients that were admitted in a period (which length depends on the cardinality of the admissions) where at least 90% of patients’ records were complete. Centers with a reported occupancy rate of less than 50% or with significant heterogeneity in the number of monthly admissions received queries or visits from certified monitors. After passing the above-mentioned validation system, all data from ICUs with at least four months of valid data were merged into an aggregated database.

In our study, we collected data on the included ICUs, including the number of admitted patients and geographic locations, alongside patients’ demographics, ward of origin, cause of ICU admission, and in-ICU outcomes.

### 2.3 Definitions

ICU-acquired infections were defined as infections acquired at least 48 h since admission in ICU. Only infections with microbiological confirmation and antibiotic susceptibility testing available were included in the current analysis. Each episode was diagnosed by the physician according to international guidelines (Marshall and Innes, 2003; O’Grady et al., 2011; Centers for Disease Control and Prevention [CDC], 2023) details are reported in the Supplementary materials.

All episodes of VAP, BSI, IAI and UTI occurred in the study period were included in the analysis. BSI episodes refer to primary bacteraemia, catheter-related BSI and BSI secondary to another focus of infection. IAI episodes refer to primary, secondary, tertiary and post-surgical peritonitis, infected pancreatitis, cholecystitis, cholangitis, and intra-abdominal abscesses.

For each site of infection, data were collected and analyzed uniquely for the first episode during the ICU stay. First episode of infection was defined as an infection not active at the time of admission, according to CDC/NHSN definition (Centers for Disease Control and Prevention [CDC], 2023).

CRPA was defined as *Pseudomonas aeruginosa* resistant to meropenem or ertapenem; similarly we identified as carbapenem-resistant any *Klebsiella* and *Acinetobacter* species isolate that did not show susceptibility to at least one carbapenem drug.

### 2.4 Statistical analysis

Continuous variables were summarized with mean and standard deviation, while categorical data were presented as counts and percentages.

The presence of any trend in the proportions of infections and of CR-GNB in different sites during the years was tested with a binomial regression, utilizing a stepwise selection methodology for optimal model fitting. Additionally, trends in the incidence rates of different HAIs, namely VAP or BSI, were examined over time. A Poisson distribution was assumed for infection counts, and a Poisson regression model was applied with exposure time considered as an offset, while the year of observation served as the independent variable. To capture temporal patterns, orthogonal polynomials were utilized. To assess monotonic and U-shaped dependence, we tested polynomials of degree one and two. If the second order was significant, we also tested higher degrees, selecting the maximum degree through a forward procedure, using a log-likelihood ratio test at a significance level of 0.01. The findings were presented using 95% confidence bands.

### 2.5 Ethics

The PROSAFE study protocol was approved by the local ethics committees at the participating centers. Written informed consent for use of clinical data was obtained according to national regulations.

## 3 Results

### 3.1 Study population

Data from 299,280 patients admitted to the involved Italian ICUs between 2013 and 2022 were included in the analysis. Patients were predominantly male (180477/299280, 60.3%) and aged 65 years or older (180477/299280, 60%). Details on the study cohort are depicted in Supplementary Table 1. Mean ICU stay was 6 ± 10 days, and, during this period, 5.9% (17678/299280) of patients had a microbiologically confirmed infection (Table 1). Among these patients, more than a half (9405/17678, 53.2%) were referred directly from the Emergency Department, while approximately one-fifth (3401/17678, 19.2%) came from surgical wards and 12% from both medical wards (2050/17678, 11.6%) and other ICUs (2191/17678, 12.4%). Two thirds of the patients, on average, reported trauma (11826/17678, 66.9%). Median ICU stay was 18 days (IQR 11–28) and overall intra-ICU mortality exceeded 20% (3572/17678, 20.2%).

**TABLE 1:** Characteristics and intensive care unit outcomes of 17,678 patients with a documented healthcare-associated infection.

Variable (%)	Total (Pt = 17678)	2013 (Pt = 1818)	2014 (Pt = 1865)	2015 (Pt = 1676)	2016 (Pt = 1865)	2017 (Pt = 1979)	2018 (Pt = 1856)	2019 (Pt = 2050)	2020 (Pt = 1319)	2021 (Pt = 1600)	2022 (Pt = 1650)
Male sex	12027 (68.0%)	1235 (67.9%)	1239 (66.4%)	1144 (68.3%)	1230 (66.0%)	1344 (67.9%)	1297 (69.9%)	1415 (69.0%)	900 (68.2%)	1087 (67.9%)	1136 (68.8%)
Age > 65	9143 (51.7%)	941 (51.8%)	1020 (54.7%)	897 (53.5%)	1001 (53.7%)	1069 (54.0%)	974 (52.5%)	1004 (49.0%)	667 (50.6%)	773 (48.3%)	797 (48.3%)
Trauma before ICU admission	11826 (66.9%)	1215 (66.8%)	1273 (68.3%)	1133 (67.6%)	1274 (68.3%)	1341 (67.8%)	1208 (65.1%)	1342 (65.5%)	866 (65.7%)	1060 (66.2%)	1114 (67.5%)
ICU LOS (Mean ± SD)	22 ± 17	22 ± 17	22 ± 18	22 ± 17	21 ± 16	21 ± 16	22 ± 18	21 ± 16	22 ± 17	21 ± 16	22 ± 16
**Source ward**											
Medical	2050 (11.6%)	210 (11.6%)	218 (11.7%)	198 (11.9%)	225 (12.1%)	236 (12.0%)	231 (12.5%)	229 (11.2%)	145 (11.1%)	190 (11.9%)	168 (10.2%)
Surgical	3401 (19.3%)	399 (22.0%)	437 (23.5%)	357 (21.4%)	351 (18.9%)	379 (19.2%)	299 (16.2%)	395 (19.4%)	212 (16.2%)	283 (17.7%)	289 (17.6%)
Emergency	9405 (53.4%)	974 (53.8%)	933 (50.2%)	859 (51.5%)	1003 (53.9%)	1026 (52.1%)	987 (53.4%)	1102 (54.1%)	721 (55.0%)	853 (53.5%)	947 (57.6%)
Other ICU	2191 (12.4%)	166 (9.2%)	213 (11.5%)	194 (11.6%)	222 (11.9%)	259 (13.1%)	270 (14.6%)	248 (12.2%)	196 (14.9%)	232 (14.5%)	191 (11.6%)
High dependency unit	561 (3.2%)	63 (3.5%)	59 (3.2%)	61 (3.7%)	60 (3.2%)	70 (3.6%)	60 (3.2%)	64 (3.1%)	38 (2.9%)	37 (2.3%)	49 (3.0%)
**ICU-outcomes**											
Death	3572 (20.2%)	373 (20.5%)	383 (20.5%)	337 (20.2%)	417 (22.4%)	438 (22.2%)	394 (21.3%)	383 (18.7%)	235 (17.8%)	310 (19.4%)	302 (18.4%)
Transferred within same hospital	9700 (55.0%)	1001 (55.1%)	1049 (56.2%)	955 (57.2%)	1018 (54.7%)	1086 (55.0%)	1015 (54.8%)	1129 (55.2%)	705 (53.4%)	852 (53.4%)	890 (54.1%)
Transferred to other hospital	4186 (23.7%)	428 (23.5%)	410 (22.0%)	362 (21.7%)	410 (22.0%)	429 (21.7%)	418 (22.6%)	510 (24.9%)	359 (27.2%)	423 (26.5%)	437 (26.6%)
Discharged home	71 (0.4%)	8 (0.4%)	6 (0.3%)	7 (0.4%)	9 (0.5%)	10 (0.5%)	5 (0.3%)	9 (0.4%)	5 (0.4%)	5 (0.3%)	7 (0.4%)
Palliative care	120 (0.7%)	8 (0.4%)	17 (0.9%)	10 (0.6%)	7 (0.4%)	13 (0.7%)	19 (1.0%)	16 (0.8%)	15 (1.1%)	7 (0.4%)	8 (0.5%)

Pt: total number of patients admitted to intensive care unit; ICU, intensive care unit; LOS, length of stay; SD, standard deviation.

### 3.2 Overall burden of gram-negative and carbapenem-resistant infection

Over the 10-year study period, 25,966 microbiologically confirmed HAIs were diagnosed by physicians in the included Italian ICUs, with an average of 1.5 infections per patient.

**FIGURE 1:**
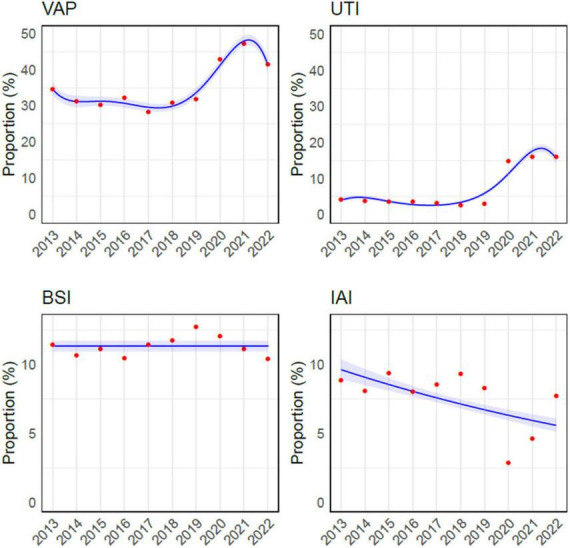
Overview of infections acquired in intensive care unit during the study period. The red dots are the observed values, while the curves had been obtained through Binomial approximation. VAP, ventilator-associated pneumonia; UTI, urinary tract infection; BSI, bloodstream infection; IAI, intrabdominal infection.

The total number of infections per year was the lowest in 2019 and 2022 (2239 and 2280 episodes respectively) and the highest in 2020 and 2021 (2889 and 3527 episodes); in the remaining years the number of ICU acquired infections was stable between 2300 and 2700 episodes each year. On average the number of HAIs per patient was always higher than 1.0 for the whole study period and, the highest number of HAIs per patient were recorded in 2020 and 2021 (2.19 and 2.21 HAIs per patient respectively), while 2019 was the year with the lowest rates (1.09 HAIs per patient). Further details on HAIs characteristics and distribution are depicted in Figure 1 and Supplementary Table 2.

**FIGURE 2:**
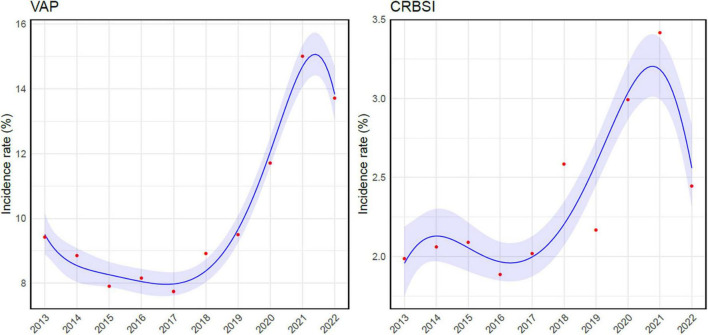
Poisson regression analysis correlating ventilator-associated pneumonia and catheter-related bloodstream infection incidence rates and time. VAP, ventilator-associated pneumonia; CRBSI, catheter-related bloodstream infection.

During the study period, VAP, BSI, IAI, and UTI accounted for 16,080 episodes (61.9%), and VAP was the most common HAI, representing more than one-third of infections (9260/25966, 35.6%), while BSI were the second most frequent, with 2,940 episodes (2940/25966, 11.3%). The incidence of VAP ranged from a minimum of 7.8/1000 mechanical ventilation days (MV, C.I. 7.2– 8.3) to a maximum of 15/1000 MV days (C.I. 14.3– 15.7), reported in 2017 and 2021 respectively. The rate of BSI for those with an indwelling venous catheter varied from 1.9/1000 catheter-days (C.I. 1.7– 2.1) in 2016 to 3.4/1000 catheter-days (C.I. 3.1– 3.7) in 2021. The Poisson regression analysis revealed a statistically significant upward trend over the years in both VAP (p-value = < 0.001) and catheter related-BSI (p-value < 0.001) incidence rates (Figure 2).

Almost half of the infections acquired in ICU (12060/25966, 46.5%) were attributed to the three GNBs studied (*Pseudomonas aeruginosa*, *Klebsiella* and *Acinetobacter* spp.), with *Klebsiella* spp. being the most frequently isolated overall (5059/25966, 19.5%), and in all four sites of infection considered, followed by *Pseudomonas aeruginosa* (4818/25966, 18.6%) and *Acinetobacter* spp. (2183/25966, 8.4%). Overall, almost a quarter (2927/12060, 24.3%) of all *Pseudomonas aeruginosa*, *Klebsiella* spp. and *Acinetobacter* spp. strains were resistant to carbapenems, with *Klebsiella* spp. expressing most frequently this susceptibility profile (1588/5059, 31.4%), followed by *Pseudomonas aeruginosa* (1049/4818, 21.8%) and *Acinetobacter* spp. (290/2183, 13.3%).

The percentage of GNB infections caused by carbapenem-resistant strains peaked in the years 2013 and 2015 surpassing one in every four infections episodes (27.3% and 27.0% respectively) and then steadily decreased until the year 2018 (20.1%). After that, a new increase in the prevalence of CR-GNBs was observed in 2020, with 24.3% of GNBs isolates displaying resistance to carbapenems. A following decrease was reported in 2021 and 2022 (22.5% and 22.7% respectively).

Among the GNB-associated infections analyzed, VAP was the most frequently reported syndrome, accounting for 20.6% of the episodes (5350/25966), followed by IAI and BSI (920 and 837 episodes, 3.5% and 3.2% respectively), while UTI was less frequent (643, 2.5%).

Carbapenem-resistant pathogens were the causative agent in almost one-third of all IAIs caused by GNBs (303/920, 32.9%) and in one-quarter each for BSIs, UTIs and VAPs (27.4, 25.3 and 24.4% respectively).

**FIGURE 3:**
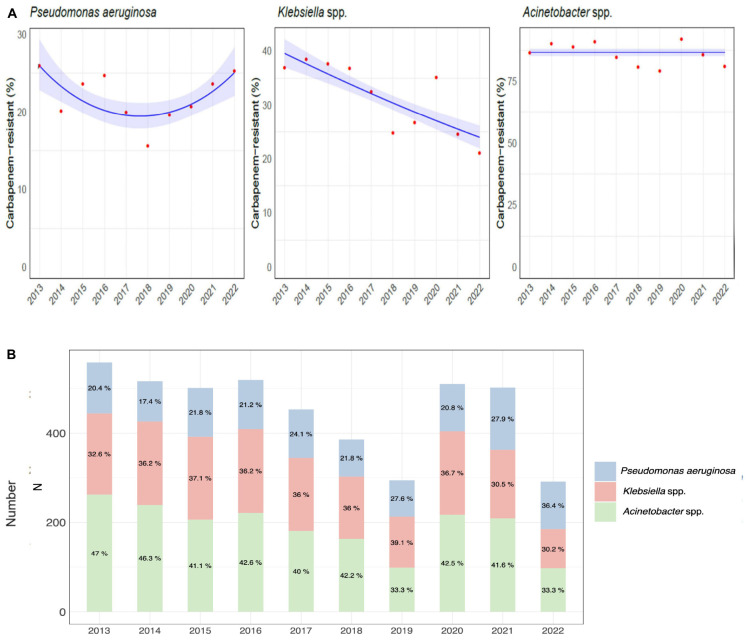
Etiology of hospital-acquired infections caused by carbapenem-resistant gram-negative bacteria over the course of the study. **(A)** Trend models of the carbapenem-resistance of *Pseudomonas aeruginosa*, *Klebsiella* spp., and *Acinetobacter* spp. **(B)** Distribution and percentage of hospital-acquired infections caused by carbapenem-resistant *Pseudomonas aeruginosa*, *Klebsiella*, and *Acinetobacter* species.

A significant increasing trend over time was observed for *Pseudomonas aeruginosa* and for *Klebsiella* spp. (U-shaped, *p*-value < 0.01 and linear, *p*-value < 0.001, respectively), while there was no significant trend for *Acinetobacter* (Figure 3A).

The distribution of CR-GNB infections by species during the study period is shown in Figure 3B and detailed in Supplementary Table 2.

#### 3.2.1 *Pseudomonas aeruginosa* infections

*Pseudomonas aeruginosa* was isolated in 18.6% of infections diagnosed (4818/25966) and displayed resistance to carbapenems in 21.8% of the cases (1049/4818). Almost one-quarter of VAP were caused by *Pseudomonas aeruginosa* (2055/9260, 22.2%), followed by IAI (361/1921, 18.8%), UTI (269/1959, 13.7%) and BSI (223/2940, 7.6%). The highest rates of carbapenem resistance were observed in IAI (109/361, 30.2%) and VAP (487/2055, 23.7%), while UTI and BSI were caused by CRPA in 16% (44/269) and 22% (49/223) of episodes, respectively. CRPA rates peaked between 2013 and 2016 (26% and 24.7% of all ICU-acquired infections, respectively) and gradually decreased in the following years reaching a nadir of 15.6% in 2018; thereafter, a progressive increase was observed, with CRPA rates reaching 20.7%, 23.6% and 25.3% in the years between 2020 and 2022.

A statistically significant correlation between CRPA infection and VAP over time was observed (*p*-value < 0.01), while no statistically significant evidence was found for other infection sites (Supplementary Figure 1).

Notably, in 2022, CRPA rates were higher than any other CR-GNBs (48.8% of all infections caused by carbapenem-resistant strains).

**FIGURE 4:**
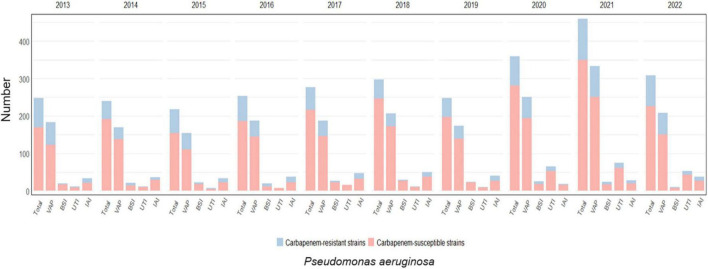
Distribution of infections caused by *Pseudomonas aeruginosa* acquired in intensive care during the study period, overall and for infection site, with the prevalence of carbapenem-resistant strains.

The trend for *Pseudomonas aeruginosa* infections and the prevalence of carbapenem-resistance per year and by infection site is depicted in Figure 4 and detailed in Supplementary Table 3.

#### 3.2.2 *Klebsiella spp*. infections

*Klebsiella* spp. was responsible for 19.5% of the total infections acquired during ICU stay (5059/ 25966) showing the highest rates of resistance to carbapenems between the GNBs analyzed, with almost one every three *Klebsiella* spp. isolates being not susceptible to this drug class (1588/ 5059, 31.4%).

Furthermore, *Klebsiella* spp. was the most common GNB in all four infections sites considered, particularly in VAP (2149/9260, 23.2%) followed by IAI (388/1921, 20.2%), BSI (467/2940, 15.9%) and UTI (297/1959, 15.2%). The highest rate of resistance to carbapenems was observed in IAI, where almost one every two isolates displayed this phenotype (180/388, 46.4%); although still representing more than one third of cases, the rates were lower for UTI (109/297, 36.7%), BSI (161/467, 34.5%) and VAP (687/2149, 32%).

The highest carbapenem-resistance rates were observed between the years 2013 and 2016, always above 35%, peaking in 2014 when 38.5% of all *Klebsiella* spp. isolates were resistant to carbapenems. In the following years rates decreased steadily until 2018, when resistance rates were 24.7%, with a subsequent peak registered in 2020 (35.1%).

Over the years, a decreasing trend was observed for carbapenem-resistant *Klebsiella* species for VAP (*p*-value < 0.001) and UTI (*p*-value < 0.003), while the trend was not significant for BSI (*p*-value = 0.134) and IAI (*p*-value = 0.018). The trend is depicted in Supplementary Figure 2.

**FIGURE 5:**
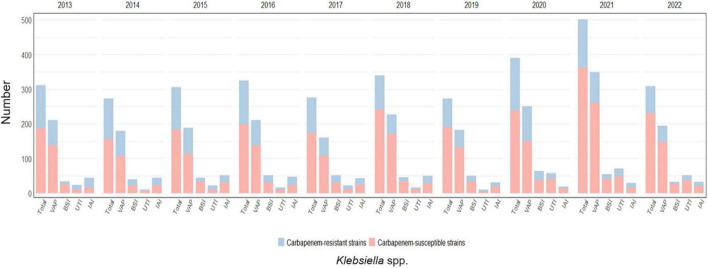
Distribution of infections caused by *Klebsiella* spp. acquired in intensive care during the study period, overall and for infection site, with the relative prevalence of carbapenem-resistant strains.

*Klebsiella* spp. infections and the prevalence of carbapenem-resistance per year and by infection site is shown in Figure 5 and detailed in Supplementary Table 4.

#### 3.2.3 *Acinetobacter* spp. infections

*Acinetobacter* spp. was the least frequently isolated GNB of the three in study (2,183/25,966, 8.4%) and displayed the overall highest rates of resistance to carbapenems (1,893/2,183, 86.7%). This pathogen was mainly responsible for VAP (1146/9260, 12.4%) and to a lesser extent for IAI (171/1,921, 9.0%), BSI (147/2,940, 5.0%), and UTI (77/1959, 3.9%). The carbapenem resistance proportion was the highest in IAI (157/171, 91.8%), followed by UTI (70/77, 90.1%), VAP (1,016/1146, 88.7%), and BSI (130/147, 88.4%). Carbapenem-resistant *Acinetobacter* spp. rates varied greatly from one year to the other, with a peak of 91.9% in 2020.

**FIGURE 6:**
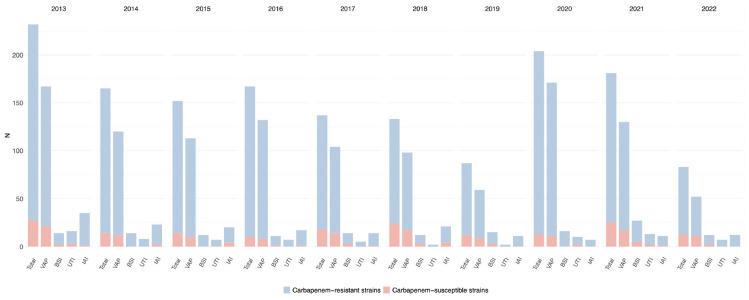
Distribution of infections caused by *Acinetobacter* spp. acquired in intensive care during the study period, overall and for infection site, with the relative prevalence of carbapenem-resistant strains.

This fact was confirmed by the tests for correlation between the presence of carbapenem-resistant *Acinetobacter* spp. in every infection site and time, that did not show significant results (*p*-value = 0.349 for VAP, *p*-value = 0.237 for BSI, *p*-value = 0.931 for UTI, and *p*-value = 0.188 for IAI), see Supplementary Figure 3. The trend for *Acinetobacter* spp. infections and the prevalence of carbapenem-resistance per year and by infection site is depicted in Figure 6 and detailed in Supplementary Table 5.

## 4 Discussion

The findings of our study shed light on the epidemiology of HAIs in Italian ICUs over a ten-year period. Our analysis reveals a substantial burden of HAIs, with an average of 1.5 infections per patient over the study period, with high prevalence of CR-GNB, particularly *Pseudomonas aeruginosa*, *Klebsiella* and *Acinetobacter* species. This trend was mainly driven by *Klebsiella* spp. and *Pseudomonas aeruginosa*, with 31.4% and 21.8% of isolate showing this susceptibility profile, respectively. In particular, CR-GNB accounted for a third of IAI and a quarter of each VAP, BSI and UTI caused by these pathogens. Notably, over the course of the decade, up to 90% of *Acinetobacter* spp. isolates retrieved showed carbapenem-resistant. Finally, during the SARS-CoV-2 pandemic, ICU-HAIs showed a peak in both incidence and CR-GNB rates, in contrast to a previously declining trend.

The burden of CR-GNBs in ICUs represents a pressing public health concern, as these facilities serve as epicenters for the convergence of critically ill patients, invasive procedures, and frequent antibiotic use, creating an environment ripe for the proliferation of resistant organisms and conditioning the patient outcomes. While rare in other GNB such as *Escherichia coli*, carbapenem resistance in strains of *Pseudomonas aeruginosa*, *Klebsiella* and *Acinetobacter* species has reached worrying percentages in Europe, especially in southern countries such as Italy, Spain, and Greece. Data from the ECDC latest surveillance report (European Centre for Disease Prevention and Control, 2022) showed indeed an increase in the population weighted mean MDR percentage over the period 2018–2022 and an increasing trend in infections caused by CRPA and carbapenem-resistant *Klebsiella* and *Acinetobacter* spp. In particular, *Klebsiella pneumoniae* showed the largest increase against all other bacterial species. Our study confirms how these findings, collected from European hospitals regardless of the intensity of care, are even amplified when focusing on the ICU setting, especially in a country like Italy where MDR prevalence remains notably high. In our cohort, up to half of all ICU-acquired infections were caused by *Pseudomonas aeruginosa*, *Klebsiella* and *Acinetobacter* species, with one every four strains resulting carbapenem-resistant. Notably, the total number of ICU-acquired infections was the highest in the years 2020 and 2021, concomitantly with the first waves of the SARS-CoV-2 pandemic, and VAP was the most common infection, accounting for one third of all HAI and almost doubling its incidence rate when compared to the pre-pandemic periods. Similarly, we observed a steadily decreasing trend of prevalence of CR-GNB infections from 2015 to 2018, followed by a new increase in 2020. The unprecedented threat posed to ICU during the SARS-CoV-2 pandemic indeed provided an ideal landscape for the development of infectious complications and spread of MDR strains (Grasselli et al., 2021; Mangioni et al., 2023a) and may serve as a crucial example for the management of future viral pandemics. The large numbers of patients requiring enhanced care, combined with the need for ICU beds and the creation of new emergency facilities, severely limited infection control practices, favoring outbreaks and the spread of nosocomial pathogens.

This global trend was confirmed in all three GNBs analyzed in our cohort.

More than a fifth (21.8%) of *Pseudomonas aeruginosa* infections were caused by carbapenem-resistant strains, in line with the latest European data that show a global CR rate of 18%, with a slight decrease observed in Italian hospitals, where the resistance to carbapenems decreases to 16% (European Centre for Disease Prevention and Control, 2022). It is noteworthy that three-quarters of Italian isolates analyzed in the report come from a setting other than the ICU, possibly accounting for the difference in MDR proportion with our cohort. Regarding the impact of the SARS-CoV-2 pandemic, we observed a concomitant new peak in CRPA rates, which previously showed a decreasing trend until 2018. Alarmingly, in 2022, the prevalence of CRPA surged to nearly half of all HAIs caused by CR-GNB. Considering the scarce therapeutic options available against this pathogen and the diverse array of resistance mechanisms it harbors, if this data is confirmed in the coming years, it will pose a significant menace to ICUs.

*Klebsiella* spp. was the most common retrieved pathogen, both overall and by site of infection, and carbapenem-resistant strains were present in almost a third of all HAIs, in line with the Italian data gathered from the European surveillance (26–29%) (European Centre for Disease Prevention and Control, 2022), causing up to a quarter of all VAP. After a peak in the years 2013–2016, when resistance rates were close to 40%, carbapenem-resistant strains decreased steadily until the pre-SARS-CoV-2 pandemic, and then showed a new increase from 24.7% to a worrying 35.1%, in line with the data from the early years of the 2010 decade.

In our cohort, *Acinetobacter* spp. caused less than one tenth of all ICU-acquired infections and showed the overall highest rates of resistance to carbapenems (86.7%). This data is in line with European and national reports, where carbapenem-resistant strains account for up to one third of all isolates globally, with even higher percentages in Italy, where carbapenem-resistance in *Acinetobacter baumannii* reaches peaks of 88% (European Centre for Disease Prevention and Control, 2022). These findings confirm an alarmingly high prevalence of carbapenem-resistant strains in infections among critically ill patients, a trend previously observed in other European studies outside the ICU (Said et al., 2021; Kinross et al., 2022). As showed by our data, infections caused by *Acinetobacter* spp. typically exhibit a varied distribution, marked by sporadic outbreaks, thereby serving as an indicator for evaluating infection control and prevention strategies. The emergence of the SARS-CoV-2 pandemic has accentuated these distinctive patterns, highlighting avenues for enhancing management approaches (Mangioni et al., 2023b).

Notably, IAI showed the highest prevalence of CR-GNB among the infectious syndromes studied, with one in three episodes caused by carbapenem-resistant strains. High rates of carbapenem-resistance in hospital-acquired IAI have already been reported (Liu et al., 2020) and may be explained by the characteristics of critically ill patients suffering from these conditions, with a higher need for invasive maneuvers and indwelling devices, such as drainages, and undergoing surgery on a non-sterile body site.

The study has several limitations that should be acknowledged. First, it relies on retrospective data collected from electronic records, which may be subject to inaccuracies or missing information. Moreover, the retrospective nature of the study limits the ability to establish causality or infer temporal relationships. Our study focuses exclusively on Italian ICUs, limiting the generalizability of the findings to other healthcare settings or regions. Due to its multicentric design, the study did not provide comprehensive data regarding antimicrobial stewardship practices, infection control measures, and individual patient factors, all of which could potentially influence the prevalence and outcomes of infections. Finally, the major limitation of our study was the lack of in-depth clinical data characterizing the infectious episodes described and the effect of MDR on the patients’ outcomes. Further analysis to address these research questions are ongoing on this cohort.

Our study also shows several strengths. Firstly, its multicentric design allowed to gather data from ICUs all over Italy, analyzing almost three hundred thousand patients. Secondly, data quality was guaranteed by the strict surveillance on its collection thanks to a validation system, and only the centers with high quality data collection were allowed in the final analysis. Thirdly, only ICU-acquired infections with microbiological confirmation and antibiotic susceptibility analysis available were analyzed, which guaranteed a better characterization of isolates and reinforced the clinical diagnosis. This choice might have resulted in an underestimation of the prevalence of CR-GNB, as it disregarded colonization (such as *Acinetobacter* spp.) and excluded instances where microbiological investigations were not conducted or yielded incomplete results. Finally, our observations spanned over a ten-year period, encompassing both the periods before and during the SARS-CoV-2 pandemic, allowing to understand its impact on the rate of HAIs and particularly on infections caused by CR-GNBs.

In conclusion, our study underscores the escalating threat of MDR infections in ICU settings, exacerbated by the SARS-CoV-2 pandemic. Addressing this challenge, particularly in anticipation of potential future viral pandemics, requires a multifaceted strategy. This approach should encompass rigorous implementation of infection control measures, antimicrobial stewardship programs, and collaborative efforts across healthcare sectors to safeguard patient safety, preserve the efficacy of antimicrobial therapies and mitigate the spread of MDR pathogens.

## Data availability statement

The original contributions presented in the study are included in the article/Supplementary material, further inquiries can be directed to the corresponding authors.

## Ethics statement

The studies involving humans were approved by the Local ethics committees at the participating centers. The studies were conducted in accordance with the local legislation and institutional requirements. Written informed consent for participation was not required from the participants or the participants’ legal guardians/next of kin in accordance with the national legislation and institutional requirements.

## Author contributions

GS: Conceptualization, Writing–original draft, Writing–review and editing, Methodology, Supervision, Validation, Visualization. MP: Conceptualization, Data curation, Formal analysis, Methodology, Writing–review and editing. MC: Conceptualization, Data curation, Investigation, Methodology, Supervision, Visualization, Writing–original draft, Writing–review and editing. CG: Conceptualization, Supervision, Visualization, Writing–original draft, Writing–review and editing. FB: Writing–original draft. AC: Conceptualization, Supervision, Writing–review and editing. BV: Supervision, Validation, Writing–review and editing. AB: Supervision, Writing–review and editing. AG: Conceptualization, Funding acquisition, Investigation, Resources, Supervision, Visualization, Writing–review and editing. SF: Conceptualization, Data curation, Formal analysis, Methodology, Supervision, Writing–original draft, Writing–review and editing. EP: Supervision, Visualization, Writing–original draft, Writing–review and editing.
