# Corrigendum: Understanding the burden of antibiotic resistance: a decade of carbapenem-resistant Gram-negative bacterial infections in Italian intensive care units

**DOI:** 10.3389/fmicb.2025.1584655

**Published:** 2025-03-18

**Authors:** Giovanni Scaglione, Matilde Perego, Marta Colaneri, Camilla Genovese, Fabio Brivio, Alice Covizzi, Bruno Viaggi, Alessandra Bandera, Andrea Gori, Stefano Finazzi, Emanuele Palomba

**Affiliations:** ^1^Department of Infectious Diseases, Luigi Sacco Hospital, Milan, Italy; ^2^Department of Biomedical and Clinical Sciences “L. Sacco”, University of Milan, Milan, Italy; ^3^Laboratory of Clinical Data Science, Department of Public Health, Mario Negri Institute for Pharmacological Research IRCCS, Ranica, Italy; ^4^Centre for Multidisciplinary Research in Health Science (MACH), University of Milan, Milan, Italy; ^5^Department of Anaesthesiology, Neuro-Intensive Care Unit, Careggi University Hospital, Florence, Italy; ^6^Department of Pathophysiology and Transplantation, University of Milano, Milan, Italy; ^7^Infectious Diseases Unit, IRCCS Ca' Granda Ospedale Maggiore Policlinico Foundation, Milan, Italy

**Keywords:** epidemiology, multidrug-resistant, intensive care unit, gram-negative, carbapenem-resistant, hospital-acquired infections

In the published article, there was an error in Figure 3, page 6 as published.

In Panel B of Figure 3 the percentage of carbapenem-resistant strains was not correct.

The corrected Figure 3 and its caption appear below.

**Figure 3:**
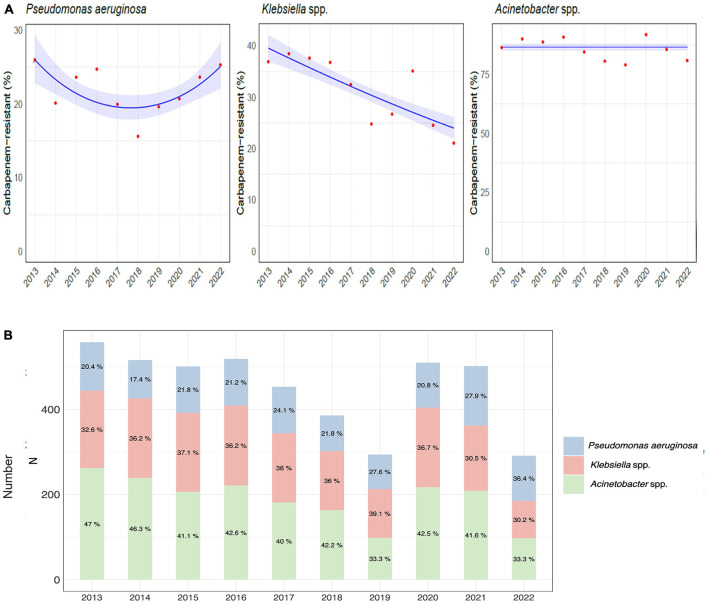
Etiology of hospital-acquired infections caused by carbapenem-resistant gram-negative bacteria over the course of the study. **(A)** Trend models of the carbapenem-resistance of *Pseudomonas aeruginosa, Klebsiella* spp., and *Acinetobacter* spp. **(B)** Distribution and percentage of hospital-acquired infections caused by carbapenem-resistant *Pseudomonas aeruginosa, Klebsiella*, and *Acinetobacter* species.

In the published article, there was an error in Figure 6, page 8 as published.

In Figure 6 the percentage of carbapenem-resistant strains was not correct.

The corrected Figure 6 and its caption appear below.

**Figure 6:**
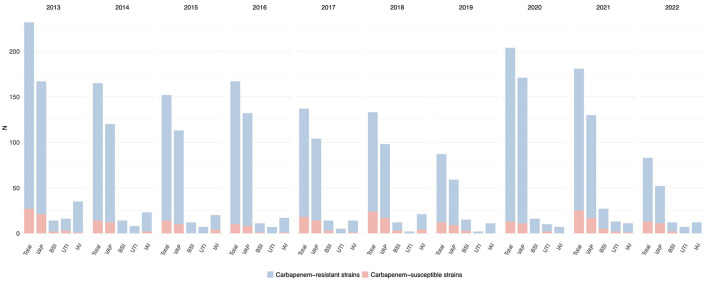
Distribution of infections caused by *Acinetobacter* spp. acquired in intensive care during the study period, overall and for infection site, with the relative prevalence of carbapenem-resistant strains.

In the published article, there was an error in **Supplementary Table 2**. The percentages of carbapenem-resistant *Acinetobacter* spp. strains were not correct. The corrected supplementary table has been published in the original article.

In the published article, there was an error in **Supplementary Table 5**. The percentages of carbapenem-resistant *Acinetobacter* spp. strains were not correct. The corrected supplementary table has been published in the original article.

In the published article, there was an error. The percentages of carbapenem-resistant *Acinetobacter* spp. strains were not correct.

A correction has been made to **Results**, *3.2.3 Acinetobacter spp. Infections*, page 7.

This sentence previously stated:

“*Acinetobacter* spp. was the least frequently isolated GNB of the three in study (2183/25966, 8.4%) and displayed the overall lowest rates of resistance to carbapenems (290/2183, 13.3%). This pathogen was mainly responsible for VAP (1146/9260, 12.4%) and to a lesser extent for IAI (171/1921, 9.0%), BSI (147/2940, 5.0%), and UTI (77/1959, 3.9%). The carbapenem resistance proportion was the highest in BSI (19/147, 13.0%), followed by VAP (130/1146, 11.3%), UTI (7/77, 9.1%) and IAI (14/171, 8.2%). Carbapenem-resistant *Acinetobacter* spp. rates varied greatly from one year to the other and this pathogen was most prevalent in the years 2019 (21%) and 2022 (19.2%)”

The corrected sentence appears below:

“*Acinetobacter* spp. was the least frequently isolated GNB of the three in study (2,183/25,966, 8.4%) and displayed the overall highest rates of resistance to carbapenems (1,893/2,183, 86.7%). This pathogen was mainly responsible for VAP (1146/9260, 12.4%) and to a lesser extent for IAI (171/1,921, 9.0%), BSI (147/2,940, 5.0%), and UTI (77/1959, 3.9%). The carbapenem resistance proportion was the highest in IAI (157/171, 91.8%), followed by UTI (70/77, 90.1%), VAP (1,016/1146, 88.7%), and BSI (130/147, 88.4%). Carbapenem-resistant *Acinetobacter* spp. rates varied greatly from one year to the other, with a peak of 91.9% in 2020.”

A correction has been made to **Discussion**, page 7.

This sentence previously stated:

“The findings of our study shed light on the epidemiology of HAIs in Italian ICUs over a ten-year period. Our analysis reveals a substantial burden of HAIs, with an average of 1.5 infections per patient over the study period, with high prevalence of CR-GNB, particularly *Pseudomonas aeruginosa, Klebsiella* and *Acinetobacter* species. This trend was mainly driven by *Klebsiella* spp. and *Pseudomonas aeruginosa*, with 31.4% and 21.8% of isolate showing this susceptibility profile, respectively. In particular, CR-GNB accounted for a third of IAI and a quarter of each VAP, BSI and UTI caused by these pathogens. Finally, during the SARS-CoV-2 pandemic, ICU-HAIs showed a peak in both incidence and CR-GNB rates, in contrast to a previously declining trend.”

The corrected sentence appears below:

“The findings of our study shed light on the epidemiology of HAIs in Italian ICUs over a ten-year period. Our analysis reveals a substantial burden of HAIs, with an average of 1.5 infections per patient over the study period, with high prevalence of CR-GNB, particularly *Pseudomonas aeruginosa, Klebsiella* and *Acinetobacter* species. This trend was mainly driven by *Klebsiella* spp. and *Pseudomonas aeruginosa*, with 31.4% and 21.8% of isolate showing this susceptibility profile, respectively. In particular, CR-GNB accounted for a third of IAI and a quarter of each VAP, BSI and UTI caused by these pathogens. Notably, over the course of the decade, up to 90% of *Acinetobacter* spp. isolates retrieved showed carbapenem-resistant. Finally, during the SARS-CoV-2 pandemic, ICU-HAIs showed a peak in both incidence and CR-GNB rates, in contrast to a previously declining trend.”

A correction has been made to **Discussion**, page 8.

This sentence previously stated:

“In our cohort, *Acinetobacter* spp. caused less than one tenth of all ICU-acquired infections and showed the overall lowest rates of resistance to carbapenems (13.3%). This data is in contrast with European and national reports, where carbapenem-resistant strains account for up to one third of all isolates globally, with even higher percentages in Italy, where carbapenem-resistance in *Acinetobacter baumannii* reaches peaks of 88% (European Centre for Disease Prevention and Control, 2022). These differences may be explained by the higher prevalence of carbapenem-resistant strains in settings different than ICU, as other European studies have already observed (Said et al., 2021; Kinross et al., 2022). Furthermore, our analysis only included infections diagnosed by a physician and did not consider respiratory, intestinal and device colonisations, which are often characteristic of *Acinetobacter* species. Finally, we considered all *Acinetobacter* spp. strains, not focusing only on *Acinetobacter baumannii*, which may have partially lowered the overall prevalence of carbapenem-resistance. As confirmed by our findings, infections caused by *Acinetobacter* spp. typically exhibit a varied distribution, marked by sporadic outbreaks, thereby serving as an indicator for evaluating infection control and prevention strategies. The emergence of the SARS-CoV-2 pandemic has accentuated these distinctive patterns, highlighting avenues for enhancing management approaches (Mangioni et al., 2023b).”

The corrected sentence appears below:

“In our cohort, *Acinetobacter* spp. caused less than one tenth of all ICU-acquired infections and showed the overall highest rates of resistance to carbapenems (86.7%). This data is in line with European and national reports, where carbapenem-resistant strains account for up to one third of all isolates globally, with even higher percentages in Italy, where carbapenem-resistance in *Acinetobacter baumannii* reaches peaks of 88% (European Centre for Disease Prevention and Control, 2022). These findings confirm an alarmingly high prevalence of carbapenem-resistant strains in infections among critically ill patients, a trend previously observed in other European studies outside the ICU (Said et al., 2021; Kinross et al., 2022). As showed by our data, infections caused by *Acinetobacter* spp. typically exhibit a varied distribution, marked by sporadic outbreaks, thereby serving as an indicator for evaluating infection control and prevention strategies. The emergence of the SARS-CoV-2 pandemic has accentuated these distinctive patterns, highlighting avenues for enhancing management approaches (Mangioni et al., 2023b).”

The authors apologize for these errors and state that this does not change the scientific conclusions of the article in any way. The original article has been updated.

