# Supplementary material for: Understanding the burden of antibiotic resistance: a decade of carbapenem-resistant Gram-negative bacterial infections in Italian intensive care units
Source: Front Microbiol. 2024 Jun 5;15:1405390. doi: 10.3389/fmicb.2024.1405390 (PMC11188344; doi:10.3389/fmicb.2024.1405390)
Supplement: Supplementary file 1 [file Table_1.DOCX]

**Supplementary materials - Understanding the burden of antibiotic resistance: a decade of carbapenem-resistant gram-negative bacterial infections in Italian intensive care units**

**Summary**

[**Coauthors:** 2](#_Toc161691392)

[**CLINICAL DEFINITION OF SPECIFIC INFECTIONS** 6](#_Toc161691394)

[**LOWER RESPIRATORY TRACT INFECTIONS** 6](#_Toc161691395)

[**BLOODSTREAM INFECTIONS (BSI)** 7](#_Toc161691396)

[**INTRA-ABDOMINAL INFECTIONS (IAI)** 8](#_Toc161691397)

[**URINARY TRACT INFECTIONS (UTIs)** 9](#_Toc161691398)

[**Supplementary Table 1**. 10](#_Toc161691399)

[**Supplementary Table 2.** 12](#_Toc161691401)

[**Supplementary Table 3** 13](#_Toc161691402)

[**Supplementary Table 4** 14](#_Toc161691403)

[**Supplementary Table 5** 15](#_Toc161691404)

[**Supplementary Figure 1** 16](#_Toc161691405)

[**Supplementary Figure 2** 17](#_Toc161691406)

[**Supplementary Figure 3** 20](#_Toc161691407)

# **Coauthors:**

**Supplementary Coauthors.** Coauthors for the GiViTI CTS (steering committee), CDC (coordinating center) and Prosafe contributors.

**a)** **CTS**

Adalgisa Caracciolo (Ente Ecclesiastico, Ospedale Generale Regionale Miulli, Acquaviva delle fonti - BA), Chieregato Arturo (ASST Grande Ospedale Metropolitano Niguarda, Milano - MI), Cipolla Cristiana (ASST Grande Ospedale Metropolitano Niguarda, Milano - MI), Dalfino Lidia (AOU Policlinico Bari - BA), Finazzi Stefano (Laboratory of Clinical Data Sciences, Dipartimento di Epidemiologia Medica, GiViTI Coordinating Center, Mario Negri Institute for Pharmacological Research IRCCS, Ranica - BG), Fumagalli Roberto (Univerity Bicocca and ASST Grande Ospedale Metropolitano Niguarda, Milano - MI), Garbero Elena (Laboratory of Clinical Data Sciences, Dipartimento di Epidemiologia Medica, GiViTI Coordinating Center, Mario Negri Institute for Pharmacological Research IRCCS, Ranica - BG), Giugni Aimone (AUSL, Ospedale Maggiore di Bologna, Bologna - BO), Paci Giulia (Università di Bologna, Bologna - BO), Olivieri Carlo (ASL Vercelli, Vercelli - VC), Ranieri Marco (Università di Bologna, Bologna - BO), Tavola Mario (Presidente Associazione GiViTI), Zamperoni Anna (Azienda Ospedaliera Cà Foncello Aulss2 Marca Trevigiana, Treviso - TV), Vergano Marco (Ospedale San Giovanni Bosco, Torino - TO), Viaggi Bruno (Azienda Ospedaliera Universitaria Careggi, Firenze - FI).

**b)** **CDC**

Valentina Barbetta (Laboratory of Clinical Data Sciences, Dipartimento di Epidemiologia Medica, GiViTI Coordinating Center, Mario Negri Institute for Pharmacological Research IRCCS, Ranica - BG), Dore Francesca (Laboratory of Clinical Data Sciences, Dipartimento di Epidemiologia Medica, GiViTI Coordinating Center, Mario Negri Institute for Pharmacological Research IRCCS, Ranica - BG), Finazzi Stefano (Laboratory of Clinical Data Sciences, Dipartimento di Epidemiologia Medica, GiViTI Coordinating Center, Mario Negri Institute for Pharmacological Research IRCCS, Ranica - BG), Garbero Elena (Laboratory of Clinical Data Sciences, Dipartimento di Epidemiologia Medica, GiViTI Coordinating Center, Mario Negri Institute for Pharmacological Research IRCCS, Ranica - BG), Perego Matilde (Laboratory of Clinical Data Sciences, Dipartimento di Epidemiologia Medica, GiViTI Coordinating Center, Mario Negri Institute for Pharmacological Research IRCCS, Ranica - BG), Tricella Giovanni (Laboratory of Clinical Data Sciences, Dipartimento di Epidemiologia Medica, GiViTI Coordinating Center, Mario Negri Institute for Pharmacological Research IRCCS, Ranica - BG), Zanetti Michele (Laboratory of Clinical Data Sciences, Dipartimento di Epidemiologia Medica, GiViTI Coordinating Center, Mario Negri Institute for Pharmacological Research IRCCS, Ranica - BG).

**c)** **Prosafe contributors**

Abastanotti Marco ( Ospedale Di Manerbio , Manerbio - BS ), Agostini Fulvio ( A.o.u. Città Della Salute E Della Scienza Di Torino , Torino - TO ), Alquati Omar ( ASST Crema Ex Ospedale Maggiore Di Crema , Crema - CR ), Alvino Stelio ( Ospedali Riuniti Valdichiana Senese Sud , Montepulciano - SI ), Amadori Carlo ( Azienda Nord Ovest Ex 6 , Cecina - LI ), Amatu Alessandro ( Fondazione IRCCS Policlinico S. Matteo , Pavia - PV ), Anelati Daniela ( Edoardo Bassini , Cinisello Balsamo - MI ), Archi Davide ( Maggiore , Lodi - LO ), Arditi Enrico ( Ospedle Policlinico San Martino IRCCS Per L'oncologia , Genova - GE ), Azzolini Maurizio ( Ospedale Santa Maria Del Carmine Rovereto , Rovereto - TN ), Babini Maria ( Ospedale Civile Lugo , Lugo - RA ), Bagalini Giampiero ( Augusto Murri , Fermo - FM ), Balestrero Virginia ( San Giacomo Asl Al Novi Ligure , Novi Ligure - AL ), Baratta Alberto ( S. Giacomo E Cristoforo , Massa - MS ), Barattini Massimo ( Santa Maria Nuova , Firenze - FI ), Bardini Alessandro ( Ospedale Civico Carrara , Carrara - MS ), Barontini Leandro ( USL Toscana Centro , Pistoia - PT ), Bartoli Teresa ( Ospedale S. M. Annunziata , Bagno A Ripoli - FI ), Bassi Giovanni ( Ospedale Civico Carrara , Carrara - MS ), Bassi Francesco ( Edoardo Bassini , Cinisello Balsamo - MI ), Becarelli Simone ( USL Toscana Centro , Prato - PO ), Beck Eduardo ( ASST Brianza - Ospedale Di Desio , Desio - MB ), Belgiorno Nicolangela ( Istituto Clinico San Rocco Di Franciacorta , Ome - BS ), Benanti Cesare ( S.S. Cosma E Damiano , Pescia - PT ), Bendinelli Matteo ( USL Toscana Centro , Pistoia - PT ), Bernard Moira ( San Martino , Belluno - BL ), Bernasconi Mara Olga ( S. Maria Della Misericordia , Rovigo - RO ), Berta Giacomo ( Aou San Luigi Gonzaga , Orbassano - TO ), Bertacchini Sara ( Azienda Ospedaliero-Universitaria S. Anna Di Ferrara , Ferrara - FE ), Bertolini Roberta ( Aoup , Pisa - PI ), Bettelli Giacomo ( Nuovo Ospedale Civile S.agostino Estense , Modena - MO ), Bignone Paola ( S. Croce , Mondovi - CN ), Biolino Piera ( A.o.u. Città Della Salute E Della Scienza Di Torino , Torino - TO ), Biscione Roberto ( Ospedale Nuovo Santa Maria Della Scaletta , Imola - BO ), Boccalatte-Rosa Daniela Luciana ( Ospedale Provinciale Di Lucca , Lucca - LU ), Boi Maria Luisa ( Azienda Ospedaliera Brotzu , Cagliari - CA ), Bonazzi Maurizio ( Edoardo Bassini , Cinisello Balsamo - MI ), Bonfà Andrea ( USL Toscana Centro , Prato - PO ), Bonifetto Giorgio ( Ospedale Santa Maria Del Prato Feltre , Feltre - BL ), Bonizzoli Manuela ( Azienda Ospedaliero Universitaria Careggi , Firenze - FI ), Bonucci Paola ( Azienda Ospedaliero-Universitaria Senese , Siena - SI ), Brandolini Ilaria ( Policlinico Tor Vergata , Roma - ), Bresadola Francesca ( Presidio Ospedaliero Area Nord Bentivoglio-Budrio-San Giovanni Persiceto , Bentivoglio - BO ), Breschi Cesare ( Azienda Ospedali Riuniti Marche Nord Presidio Di Pesaro , Pesaro - PU ), Brunetti Iole ( Ospedle Policlinico San Martino IRCCS Per L'oncologia , Genova - GE ), Brunori Emanuela ( Ospedale Civile Macerata--Av3--Asur Marche , Macerata - MC ), Buzzetti Virginio ( San Giovanni Di Dio , Orbetello - GR ), Cabano Gian Virgilio ( Azienda Ospedaliera Ospedale Di Circolo Di Busto Arsizio , Busto Arsizio - VA ), Calamai Italo ( Ospedale San Giuseppe , Empoli - FI ), Calicchio Giuseppe ( Azienda Ospedaliera Universitaria San Giovanni Di Dio E Ruggi D'aragona , Salerno - SA ), Callea Maria Preziosa ( Ospedali Riuniti Valdichiana Senese Sud , Montepulciano - SI ), Calò Mauro Antonio ( Ospedale Civile Di Mirano , Mirano - VE ), Cancellieri Francesco ( Ospedale Maggiore, C.a. Pizzardi , Bologna - BO ), Capra Carlo ( ASST Ovest Milanese - Presidio Di Magenta - Ospedale ‘G. Fornaroli’ , Magenta - MI ), Capuccini Silvia ( Spedali Civili Di Brescia , Brescia - BS ), Caracciolo Adalgisa ( Francesco Miulli , Bari - BA ), Carli Manuela ( USL Toscana Centro , Pistoia - PT ), Carnevale Livio ( Fondazione IRCCS Policlinico S.matteo , Pavia - PV ), Carrer Sara ( ASST-Rhodense - P.O. Di Rho , Rho - MI ), Carsana Cristina ( Azienda Ospedaliera Ospedale Di Circolo Di Busto Arsizio , Busto Arsizio - VA ), Casadei Edith ( Azienda Ospedaliero-Universitaria Senese , Siena - SI ), Casadio Maria Cinzia ( Ospedale Misericordia Grosseto , Grosseto - GR ), Casagrande Lucia ( Ospedale Cà Foncello Santa Maria Dei Battuti , Treviso - TV ), Casalini Pierpaolo ( Per Gli Infermi , Faenza - RA ), Casalis Michele ( USL Toscana Nord Ovest - P.O. Piombino , Piombino - LI ), Cascianini Alessandra ( Ospedale San Donato , Arezzo - AR ), Casella Umberto ( Ao Ospedale Di Circolo Di Busto Arsizio Presidio Ospedaliero Di Saronno , Saronno - VA ), Castelli Gian Paolo ( ASST - Mantova , Mantova - MN ), Castiglione Giacomo ( Azienda Ospedalero Universitaria Policlinico ‘G. Rodolico - San Marco’ , Catania - CT ), Caviglia Enrica ( Asl 3 Genovese Presidio Ospedaliero Villa Scassi , Genova - GE ), Cecchi Viviana ( Ospedale Del Mugello , Borgo San Lorenzo - FI ), Cecchi Alessandra ( Ospedale Maggiore, C.a. Pizzardi , Bologna - BO ), Chini Giuseppe ( Ospedale Del Mugello , Borgo San Lorenzo - FI ), Ciani Andrea ( S.S. Cosma E Damiano , Pescia - PT ), Ciceri Rita ( ASST Lecco , Lecco - LC ), Cigada Marco Guido Alberto ( A.O. Fatebenefratelli E Oftalmico , Milano - MI ), Clementi Stefano ( Ospedale Di Sesto San Giovanni , Sesto San Giovanni - MI ), Coaloa Maddalena ( Ospedale Ss Annunziata , Savigliano - CN ), Colombo Riccardo ( Azienda Ospedaliera ‘Luigi Sacco’ , Milano - MI ), Corsini Walter ( Ospedale Civico Carrara , Carrara - MS ), Cottignoli Tito ( Ospedale Civile Lugo , Lugo - RA ), Covani Frigieri Francesca ( Ospedale S. M. Annunziata , Bagno A Ripoli - FI ), Crema Luciano ( Istituti Ospitalieri Di Cremona , Cremona - CR ), Da Re Dolores ( Ulss 6 Euganea , Monselice - PD ), Dal Poggetto Luigi ( S.S. Cosma E Damiano , Pescia - PT ), Dalfino Lidia ( A.u.o Policlinico , Bari - BA ), Dall' Oglio Daniela ( ASST - Mantova , Mantova - MN ), De Cristofaro Anna ( Azienda Ospedali Riuniti Marche Nord Presidio Di Pesaro , Pesaro - PU ), De Lucia Marta ( Nuovo Ospedale Degli Infermi , Ponderano - BI ), Dei Poli Marco ( Policlinico San Donato , San Donato Milanese - MI ), Della Mora Ernesto ( Ospedale Cazzavillan , Arzignano - VI ), Della Selva Andrea ( Ospedale Michele E Pietro Ferrero- , Verduno - CN ), Demozzi Enrico ( Ospedale Santa Maria Del Carmine Rovereto , Rovereto - TN ), Di Masi Pierfrancesco ( Istituto Di Ricovero E Cura A Carattere Scientifico ‘Saverio De Bellis’ , Castellana Grotte - BA ), Di Pasquale Dino Aurelio Cleto ( Felice Lotti Pontedera , Pontedera - PI ), Di Stella Roberta ( Ospedale Di Circolo Fondazione Macchi , Varese - VA ), Fabbri Emilio ( G.b Morgagni-L.pierantoni , Forlì - FC ), Fabi Maria Cristina ( Santa Croce , Fano - PU ), Facondini Francesca ( Ospedale ‘Infermi’ , Rimini - RN ), Fagoni Nazzareno ( Spedali Civili Di Brescia , Brescia - BS ), Falini Stefano ( Ospedale Misericordia Grosseto , Grosseto - GR ), Fanfani Elena ( San Giovanni Di Dio , Firenze - FI ), Faraldi Loredana ( ASST Grande Ospedale Metropolitano Niguarda , Milano - MI ), Ferretti Marcus ( Azienda Ospedaliera Ospedale S.martino E Cliniche Universitarie Convenzionate , Genova - GE ), Ferri Enrico ( Ospedale Maggiore, C.a. Pizzardi , Bologna - BO ), Fiore Gilberto ( ‘Santa Croce’ - Moncalieri - Asl To 5 , Moncalieri - TO ), Fiorenzi Marco ( Ospedali Riuniti Valdichiana Senese Sud , Montepulciano - SI ), Fogliati Claudio ( Aou San Luigi Gonzaga , Orbassano - TO ), Fognani Giuliana ( Spedali Riuniti Livorno , Livorno - LI ), Fontaneto Carlotta ( S.andrea , Vercelli - VC ), Gabini Rita ( Ospedale San Donato , Arezzo - AR ), Galante Dario ( Asl Foggia Ospedale G. Tatarella Di Cerignola , Cerignola - FG ), Galbiati Rita ( Presidio Ospedaliero ‘Armando Businco’ , Cagliari - CA ), Galeotti Elsa ( Ospedale Santa Maria Del Prato Feltre , Feltre - BL ), Galleschi Nicola ( Ospedale San Giuseppe , Empoli - FI ), Gamberini Emiliano ( Maurizio Bufalini , Cesena - FC ), Garelli Alberto ( Santa Maria Delle Croci , Ravenna - RA ), Gianni Massimo ( Ospedale Regionale Umberto Parini , Aosta - AO ), Giannoni Stefano ( Ospedale San Giuseppe , Empoli - FI ), Girardis Massimo ( Azienda Ospedaliera Universitaria Di Modena , Modena - MO ), Giudici Riccardo ( ASST Grande Ospedale Metropolitano Niguarda , Milano - MI ), Giudici Daniela ( IRCCS Ospedale San Raffaele , Milano - MI ), Giudici Riccardo ( ASST Gom Niguarda , Milano - MI ), Giuntini Romano ( Ospedale San Giuseppe , Empoli - FI ), Gorietti Adonella ( Santa Maria Della Misericordia , Perugia - PG ), Guadagnucci Alberto ( S Giacomo E Cristoforo , Massa - MS ), Guarducci Maria Diletta ( Ospedale S. M. Annunziata , Bagno A Ripoli - FI ), Guerra Emmanuele ( Nuovo Ospedale Civile S.agostino Estense , Modena - MO ), Hellmann Ferruccio ( Ospedale Ss Annunziata , Savigliano - CN ), Isetta Michele ( Asl 3 Genovese Presidio Ospedaliero Villa Scassi , Genova - GE ), Jorio Antonella ( Area Vasta 2 , Jesi - AN ), Kola Dea ( Policlinico Di Abano Terme , Abano Terme - PD ), Lanza Maria Concetta ( G.b Morgagni-L.pierantoni , Forlì - FC ), Lanza Giuseppina ( Ospedale Michele E Pietro Ferrero- , Verduno - CN ), Lefons Ugo ( Ospedale Alta Val D'elsa , Poggibonsi - SI ), Madeira Susana Monica ( Ospedale Del Casentino , Bibbiena - AR ), Madonna Roberto ( Ospedale Misericordia Grosseto , Grosseto - GR ), Magatti Mariafederica ( Azienda Ospedaliera S.anna- Ospedale S.anna , San Fermo Della Battaglia - CO ), Maitan Stefano ( Per Gli Infermi , Faenza - RA ), Malacarne Paolo ( Aoup , Pisa - PI ), Mangani Valerio ( San Giovanni Di Dio , Firenze - FI ), Mannolini Giovanni ( Ospedale ‘S.antonio Abate’ , Pontremoli - MS ), Mantovani Giorgio ( Azienda Ospedaliero-Universitaria Di Ferrara, Arcispedale S. Anna , Ferrara - FE ), Marafon Silvio ( Ospedale Cazzavillan , Arzignano - VI ), Marceddu Alessandra ( Giovanni Paolo II , Olbia - OT ), Mariconti Laura ( Maggiore , Lodi - LO ), Marini Federica ( Ospedale Alta Val D'elsa , Poggibonsi - SI ), Marino Giovanni ( Azienda Ospedaliera Di Melegnano Presidio Di Vizzolo Predabissi , Vizzolo Predabissi - MI ), Martin Marina Alessandra ( San Bortolo , Vicenza - VI ), Martino Oronzo ( Madonna Delle Grazie , Matera - MT ), Mastroianni Alessandro ( Maggiore , Chieri - TO ), Mediani Teresa Sabina ( Fondazione Policlinico San Matteo , Pavia - PV ), Melani Dario ( USL9 , Grosseto - GR ), Melis Martina ( Giovanni Paolo II , Olbia - OT ), Mengoli Francesca ( Ospedale Maggiore, C.a. Pizzardi , Bologna - BO ), Mereto Nadia ( Asl 3 Genovese - P.O. Villa Scassi , Genova - GE ), Messina Marianna ( Giovanni Paolo II , Olbia - OT ), Meucci Monica ( Ospedale Regionale Umberto Parini , Aosta - AO ), Militano Carmine Rocco ( Fondazione Poliambulanza - Istituto Ospedaliero , Brescia - BS ), Molesi Andrea ( Area Vasta 2 , Jesi - AN ), Monge Roffarello Claudia Angela ( Ospedale Ss Annunziata , Savigliano - CN ), Morosini Paolo ( Area Vasta 2 Asur Marche , Fabriano - AN ), Mucci Milena ( IRCCS Ospedale San Raffaele , Milano - MI ), Munaron Susanna ( Ospedale San Giacomo , Castelfranco Veneto - TV ), Napoleone Alessandra ( Azienda Ospedaliera Brotzu , Cagliari - CA ), Nardi Giuseppe ( Azienda Ospedaliera San Camillo Forlanini , Roma - RM ), Nardini Massimiliano ( Versilia , Lido Di Camaiore - LU ), Natalini Giuseppe ( Fondazione Poliambulanza - Istituto Ospedaliero , Brescia - BS ), Nava Luana ( Asl 3 Genovese - P.O. Villa Scassi , Genova - GE ), Negri Giovanni ( ASST Ovest Milanese - Presidio Di Magenta - Ospedale ‘G. Fornaroli’ , Magenta - MI ), Negro Giancarlo ( Francesco Ferrari , Casarano - LE ), Negro Giancarlo ( Asle Lecce - Presidio Ospedaliero Gallipoli , Gallipoli - LE ), Nicolini Andrea ( Versilia , Lido Di Camaiore - LU ), Nuovo Domenico ( A.O.U. Città Della Salute E Della Scienza Di Torino , Torino - TO ), Odetto Lorenzo ( Aou San Luigi Gonzaga , Orbassano - TO ), Olivieri Maria Candida ( Ospedale San Donato , Arezzo - AR ), Pacini Daniela Maria ( Azienda Ospedaliera Ospedale S.martino E Cliniche Universitarie Convenzionate , Genova - GE ), Padovan Lamberto ( Ulss 6 Euganea , Monselice - PD ), Parnigotto Alessandra ( Ulss 6 Euganea , Monselice - PD ), Parrini Vieri ( Ospedale Del Mugello , Borgo San Lorenzo - FI ), Pasetti Giovanni Stefano ( San Giovanni Di Dio , Orbetello - GR ), Pastorini Simonetta ( Ospedale P. Cosma-AUSL 15 Alta Padovana , Camposampiero - PD ), Pedeferri Matteo ( Azienda Ospedaliera Della Provincia Di Lecco - Presidio Ospedaliero ‘S. Leopoldo Mandic’ Merate , Merate - LC ), Pegoraro Maurizio ( Ospedale San Giacomo , Castelfranco Veneto - TV ), Pelagatti Cecilia ( Santa Maria Nuova , Firenze - FI ), Pelati Erika ( Azienda Ospedaliero Universitaria Pisana , Pisa - PI ), Pero Alice ( S.andrea , Vercelli - VC ), Pessina Carla ( ASST-Rhodense - P.O. Di Rho , Rho - MI ), Peta Mario ( Ospedale Cà Foncello Santa Maria Dei Battuti , Treviso - TV ), Petrucci Nicola ( Azienda Socio-Sanitaria Territoriale Del Garda, Presidio Di Desenzano , Desenzano Del Garda - BS ), Pezzi Angelo ( Edoardo Bassini , Cinisello Balsamo - MI ), Piccioni Giuseppe ( Spedali Civili Di Brescia , Brescia - BS ), Pignatti Alessandro ( USL Modena - Ospedle Civile 'B.ramazzini' , Carpi - MO ), Pinna Cristina ( Nuovo Ospedale Civile S.agostino Estense , Modena - MO ), Pizzali Mario ( Ospedale Civile Di Mirano , Mirano - VE ), Poole Daniele ( San Martino , Belluno - BL ), Prato Maurizio ( San Giacomo Asl Al Novi Ligure , Novi Ligure - AL ), Pulici Marco ( ASST Grande Ospedale Metropolitano Niguarda , Milano - MI ), Rambaldi Marco ( Nuovo Ospedale Civile S.agostino Estense , Modena - MO ), Randellini Roberto ( Ospedali Riuniti Valdichiana Senese Sud , Montepulciano - SI ), Righini Erminio ( Ospedale Del Delta - Azienda USL Ferrara , Lagosanto - FE ), Riva Ivano ( ASST Papa Giovanni XXIII , Bergamo - BG ), Rizzi Francesco ( Ospedale Santa Maria Del Prato Feltre , Feltre - BL ), Robbiati Alessandro ( Ao Ospedale Di Circolo Di Busto Arsizio Presidio Ospedaliero Di Saronno , Saronno - VA ), Rona Roberto ( Azienda Ospedaliera San Gerardo , Monza - MB ), Rossi Simona ( ASST-Rhodense - P.O. Di Rho , Rho - MI ), Rossi Maurizio ( ASST Lariana , Menaggio - CO ), Roticiani Valeria ( Ospedale Santa Maria Alla Gruccia , Montevarchi - AR ), Ruggeri Patrizia ( Istituti Ospitalieri Di Cremona , Cremona - CR ), Russo Emanuele ( AUSL Romagna - Ospedale M.bufalini , Cesena - ), Saccavino Erica ( ASST Brianza - Ospedale Di Desio , Desio - MB ), Salvi Giovanni ( Presidio Ospedaliero Di Imperia , Imperia - IM ), Sambuco Monica ( Ospedale Di Circolo Fondazione Macchi , Varese - VA ), Savioli Monica ( Fondazione IRCCS Ca Granda Ospedale Maggiore Policlinico , Milano - MI ), Savioli Monica ( Fondazione IRCCS Ca Granda Ospedale Maggiore Policlinico , Milano - MI ), Scarrone Silvia ( ‘Civile - Ss Antonio E Biagio E C. Arrigo’ , Alessandria - AL ), Serio Daniela ( Asl Taranto , Taranto - TA ), Serra Luciano ( Presidio Ospedaliero ‘Armando Businco’ , Cagliari - CA ), Sgarioto Vincenzo ( ASST - Mantova , Mantova - MN ), Sicignano Alberto ( Fondazione IRCCS Ca Granda Ospedale Maggiore Policlinico , Milano - MI ), Sorgato Cristina ( Policlinico Di Abano Terme , Abano Terme - PD ), Spagarino Ermanno ( Nuovo Ospedale Degli Infermi , Ponderano - BI ), Staccioli Paola ( USL Toscana Centro , Pistoia - PT ), Storti Enrico ( Maggiore , Lodi - LO ), Tavola Mario ( Asl 3 Genovese Presidio Ospedaliero Villa Scassi , Genova - GE ), Tenio Rita ( S. Croce , Mondovi - CN ), Terzitta Marina ( G.b Morgagni-L.pierantoni , Forlì - FC ), Testa Marco ( Ospedale Ss Annunziata , Savigliano - CN ), Tinacci Silvia ( Ospedale San Giuseppe , Empoli - FI ), Tintori Davide ( Spedali Civili Di Brescia , Brescia - BS ), Tofani Rossella ( Spedali Riuniti Livorno , Livorno - LI ), Tosi Luigi ( Ospedale ‘S.antonio Abate’ , Pontremoli - MS ), Turriziani Ilaria ( Ospedale Maggiore, C.a. Pizzardi , Bologna - BO ), Vaccari Caterina ( San Giacomo Asl Al Novi Ligure , Novi Ligure - AL ), Vecchiarelli Ada ( Santa Maria Della Misericordia , Perugia - PG ), Ventura Luciana ( ‘Civile - Ss Antonio E Biagio E C. Arrigo’ , Alessandria - AL ), Venturini Elisabetta ( ‘Civile - Ss Antonio E Biagio E C. Arrigo’ , Alessandria - AL ), Vespignani Maria Giovanna ( Ospedale Nuovo Santa Maria Della Scaletta , Imola - BO ), Visconti Maria Grazia ( Ospedale A.uboldo Presidio Ospedaliero Cernusco S/N , Cernusco Sul Naviglio - MI ), Vivaldi Nicoletta ( ‘Civile - Ss Antonio E Biagio E C. Arrigo’ , Alessandria - AL ), Zampieri Giovanni ( Ospedale Civile Di Mirano , Mirano - VE ), Zani Gianluca ( Santa Maria Delle Croci , Ravenna - RA ), Zanni Vittorio ( Presidio Ospedaliero Area Nord Bentivoglio-Budrio-San Giovanni Persiceto , Bentivoglio - BO ), Zardin Michela ( ASST - Mantova , Mantova - MN ), Zompanti Valeria ( Ospedale Civile Macerata - Av3- Asur Marche , Macerata - MC ), Zonta Giandomenico ( Azienda Socio-Sanitaria Territoriale Del Garda, Presidio Di Desenzano , Desenzano Del Garda - BS ), Zoppellari Roberto ( Azienda Ospedaliero-Universitaria Di Ferrara, Arcispedale S. Anna , Ferrara - FE ), Zuccaro Francesco ( Madonna Delle Grazie , Matera - MT )

# **CLINICAL DEFINITION OF SPECIFIC INFECTIONS**

## **LOWER RESPIRATORY TRACT INFECTIONS**

- 1. **PNEUMONIA**:

Personalized from American Journal Infection Control 2008;36:309-332.

- POSSIBLE

Criterion 1: Presence of new and persistent infiltrate on chest x-ray/CT scan AND:

- Fever > 38° or leukocytes > 12,000/ml or < 4,000/ml
- Non quantitative culture from a sample of tracheal secretions
- At least two of the following:
  1. Purulent expectorate of new onset or alterations in characteristics of expectorate (colour, odour, quantity, consistency).
  2. Cough or dyspnea or tachypnea.
  3. Suggestive lung auscultation (rales or bronchial respiratory sounds), rhonchi or wheezing.
  4. Worsening of respiratory exchange (e.g. O2 desaturation or increase in request for O2 or increase in request for ventilation).

Criterion 2: Presence of new and persistent infiltrate on chest x-ray / CT scan AND:

- fever > 38°C or leukocytes > 12,000/ml or < 4,000/ml
- at least two of the following:
  1. purulent expectorate of new onset or alterations in characteristics of expectorate (colour, odour, quantity, consistency
  2. cough or dyspnea or tachypnea.
  3. suggestive lung auscultation (rales or bronchial respiratory sounds), rhonchi or wheezing.
  4. worsening of respiratory exchange (e.g. O2 desaturation or increase in request for O2 or increase in request for ventilation.
- PROBABLE / CERTAIN

Presence of new and persistent lung infiltrate AND at least 2 compatible clinical and laboratory signs:

- fever/hypothermia or leukocytosis/leukopenia
- purulent secretions of new onset and/or their modification or cough or dyspnea or tachypnea or suggestive auscultation or worsening of gas exchanges

AND 1 or more of the following criteria:

1. Positive culture, with quantitative assessment equal to/over threshold value of 10^5-6^ CFU/ml, of one or more samples of secretions collected from the trachea by the unprotected method (tracheobronchial aspirate).
2. Positive culture, with quantitative assessment equal to/over the threshold value 10^3^ CFU/ml for quantitative cultures collected by bronchial brush (Protected Specimen Brush PSB) or ≥ 104 CFU/ml for quantitative cultures collected by bronchoalveolar lavage (BAL) or mini-BAL or ≥ 103 protected distal aspiration. PN 1
3. Concordant blood culture with organisms isolated from respiratory tract secretions. PN 3
4. Positive culture of a pleural effusion with organism concordant with organisms isolated from respiratory tract secretions.
5. Evolution and abscessualization of lung focus.
6. Other certain infectious data on viruses or particular germs (e.g. Legionella from respiratory secretions, positivity of urinary antigen for *Legionella*, *Pneumococcus*, isolation of mycobacteria, detection of *P. jirovecii*, isolation of *Aspergillus* hyphae, serum conversion with significant increase in antibody titre, presence of antigens and/or viral antibodies detected in respiratory secretions, other).
   1. **VENTILATOR ASSOCIATED PNEUMONIA (VAP)** (<https://www.cdc.gov/nhsn/pdfs/pscmanual/6pscvapcurrent.pdf>)

A pneumonia where the patient is on mechanical ventilation for > 2 consecutive calendar days on the date of event, with day of ventilator placement being Day 1* AND the ventilator was in place on the date of event or the day before.

*If the ventilator was in place prior to inpatient admission, the ventilator day count begins with the admission date to the first inpatient location. If a break in mechanical ventilation occurs for at least one full calendar day, ventilator day count for ventilator association starts anew upon reintubation and/or re-initiation of mechanical ventilation.

## **BLOODSTREAM INFECTIONS (BSI)**

(CDC/NHSN surveillance definition of health care-associated infection and criteria for specific types of infections in the acute care setting, Doi: [10.1016/j.ajic.2008.03.002](https://doi.org/10.1016/j.ajic.2008.03.002))

- 1. **Primary bacteraemia of unknown origin:**

A Laboratory Confirmed Bloodstream Infection (LCBI) that is not secondary to an infection at another body site.

- Absence of a recognized focus of infection

OR

- Organism is not related to an infection at another site

Definition of laboratory-confirmed bloodstream infection (LCBI), at least 1 of the following criteria:

1. Recognized pathogen cultured from 1 or more blood cultures AND organism cultured from blood is not related to an infection at another site. (See Notes 1 and 2)
2. Patient has at least 1 of the following signs or symptoms: fever (> 38°C), chills, or hypotension and signs and symptoms AND positive laboratory results are not related to an infection at another site AND common skin contaminant (i.e., diphtheroids [*Corynebacterium* spp], *Bacillus* [not *B. anthracis*] spp, *Propionibacterium* spp, coagulase-negative *Staphylococci* [including *S. epidermidis*], viridians group *Streptococci*, *Aerococcus* spp, *Micrococcus* spp) is cultured from 2 or more blood cultures drawn on separate occasions. (See Notes 3 and 4).

Notes

1. In criterion 1, the phrase “1 or more blood cultures” means that at least 1 bottle from a blood draw is reported by the laboratory as having grown organisms (i.e., is a positive blood culture).

2. In criterion 1, the term “recognized pathogen” does not include organisms considered common skin contaminants (see criteria 2 and 3 for a list of common skin contaminants). A few of the recognized pathogens are *S. aureus*, *Enterococcus* spp, *E. coli*, *Pseudomonas* spp, *Klebsiella* spp, *Candida* spp, and others.

3. In criteria 2 and 3, the phrase “2 or more blood cultures drawn on separate occasions” means (1) that blood from at least 2 blood draws were collected within 2 days of each and (2) that at least 1 bottle from each blood draw is reported by the laboratory as having grown the same common skin contaminant organism.

- 1. **Intravascular catheter-related BSI (CR-BSI):**

(Guidelines for the Prevention of Intravascular Catheter-related Infections, doi: [10.1093/cid/cir257](https://doi.org/10.1093%2Fcid%2Fcir257))

Presence of clinical suspicion (signs of systemic inflammation as fever, hypotension, chills) AND no other apparent source of bacteraemia/fungaemia with the exception of the catheter AND one of the following criteria:

1. semiquantitative (> 15 CFU) or quantitative culture (>10^3^ CFU) of a catheter segment with a concordant (species and antibiogram) blood culture (not necessarily quantitative) performed at the same time (within 24 hours) as catheter removal
2. differential time to positivization (at least 120 minutes) for qualitative blood culture from blood drawn through the catheter and blood culture from a peripheral vein.
3. same time to positivization of quantitative blood cultures, but with ratio of ≥ 5:1 between blood culture from vascular catheter vs blood culture from peripheral vein
   1. **Secondary BSI:**

A BSI secondary to another site of infection with the following criteria:

- An NHSN site-specific definition must be met:
- One of the following scenarios must be met:
- At least one organism from the blood specimen matches an organism identified from the site specific specimen that is used as an element to meet the NHSN site-specific infection criterion AND the blood specimen is collected during the secondary BSI attribution period (infection window period + repeat infection timeframe)
- An organism identified in the blood specimen is an element that is used to meet a NHSN site specific infection criterion, and therefore is collected during the site-specific infection window period.

## **INTRA-ABDOMINAL INFECTIONS (IAI)**

Personalized from Critical Care Medicine 2003;31:2228-2237

- 1. **Primary peritonitis:**

Peritoneal infection in the absence of visceral lesions, as infected ascites, infections in peritoneal dialysis etc.

- 1. **Secondary peritonitis:**

Localized or diffuse peritoneal infection due to a solution of continuity in gastroenteric tract, as appendicular abscess, perforation of the viscera, pancreatitis, retroperitoneal infection, etc..

- 1. **Tertiary peritonitis:**

Primary or secondary peritonitis which, despite treatment, recurs as a more severe infection with development of multiorgan failure: this occurs in weak and frail patients and is the result both of failure to control the initial focus and of the lowering of patient defences.

- 1. **Post-surgical peritonitis:**

Peritonitis or abscess or pancreatitis or retroperitoneal infections such as complications of previous surgical procedures.

- 1. **Infected acute pancreatitis:**

Acute pancreatitis + positive blood culture (in the absence of other sites of infection and/or CT-scan documentation of retroperitoneal air bubbles and/or positive culture of pancreatic tissue or drainage material or material withdrawn during surgery.

## **URINARY TRACT INFECTIONS (UTIs)**

(Personalized from American Journal Infection Control 2008;36:309-332)

- Positive urinoculture load > 10^5^ or load between 10^3^ and 10^5^ in the presence of antibiotic therapy with no more than 2 species of organism.

AND

- Clinical signs compatible with UTI (fever >38°C in the absence of other causes, pyuria, cloudy urine)
- Leukocyturia (when using stick test: at least 2nd step) : ≥ 10 leukocytes/cc

**Supplementary Table 1**. Study cohort characteristics and intensive care unit outcomes

| **Number of patients** | **Total** | **2013** | **2014** | **2015** | **2016** | **2017** | **2018** | **2019** | **2020** | **2021** | **2022** |
| --- | --- | --- | --- | --- | --- | --- | --- | --- | --- | --- | --- |
|  | 299280 | 29068 | 28267 | 27585 | 33164 | 33794 | 32885 | 36897 | 25569 | 27018 | 25033 |
| **Male Sex (%)** | 180477 (60.3%) | 17252 (59.4%) | 16754 (59.3%) | 16495 (59.8%) | 19806 (59.7%) | 20050 (59.3%) | 19609 (59.6%) | 22241 (60.3%) | 15936 (62.3%) | 16920 (62.6%) | 15414 (61.6%) |
| **Age > 65 (%)** | 181382 (60.6%) | 17941 (61.7%) | 17220 (60.9%) | 17137 (62.1%) | 20481 (61.8%) | 20961 (62.0%) | 20053 (61.0%) | 22268 (60.4%) | 15094 (59.0%) | 15490 (57.3%) | 14737 (58.9%) |
| **ICU LOS (Mean ± SD)** | 6 ± 10 | 6 ± 10 | 6 ± 10 | 6 ± 10 | 6 ± 10 | 6 ± 9 | 6 ± 10 | 6 ± 9 | 8 ± 11 | 8 ± 12 | 7 ± 11 |
| **Trauma** | 40033 (13.4%) | 4178 (14.4%) | 4093 (14.5%) | 3869 (14.1%) | 4457 (13.5%) | 4464 (13.2%) | 4406 (13.4%) | 4983 (13.5%) | 2938 (11.5%) | 3293 (12.2%) | 3352 (13.4%) |
| **ICU-outcomes** | | | | | | | | | | | |
| **Death** | 52345 (17.5%) | 4948 (17.0%) | 4816 (17.1%) | 4755 (17.3%) | 5541 (16.7%) | 5766 (17.1%) | 5460 (16.7%) | 5960 (16.2%) | 5160 (20.3%) | 5510 (20.5%) | 4429 (17.8%) |
| **Transferred within same hospital** | 216139 (72.4%) | 21224 (73.1%) | 20644 (73.1%) | 19992 (72.8%) | 24349 (73.5%) | 24847 (73.6%) | 24053 (73.4%) | 27198 (74.0%) | 17417 (68.5%) | 18583 (69.0%) | 17832 (71.6%) |
| **Transferred to other hospital** | 26783 (9.0%) | 2553 (8.8%) | 2449 (8.7%) | 2416 (8.8%) | 2878 (8.7%) | 2808 (8.3%) | 2926 (8.9%) | 3260 (8.9%) | 2599 (10.2%) | 2560 (9.5%) | 2334 (9.4%) |
| **Discharged home** | 1981 (0.7%) | 232 (0.8%) | 196 (0.7%) | 180 (0.7%) | 225 (0.7%) | 195 (0.6%) | 200 (0.6%) | 211 (0.6%) | 148 (0.6%) | 177 (0.7%) | 217 (0.9%) |
| **Palliative care** | 1132 (0.4%) | 79 (0.3%) | 124 (0.4%) | 112 (0.4%) | 113 (0.3%) | 123 (0.4%) | 123 (0.4%) | 146 (0.4%) | 113 (0.4%) | 106 (0.4%) | 93 (0.4%) |
| **Previous ward** |  | | | | | | | | | | |
| **Medical** | 45817 (15.4%) | 4178 (14.4%) | 4060 (14.4%) | 3936 (14.4%) | 4733 (14.4%) | 4786 (14.3%) | 4671 (14.3%) | 5117 (14.0%) | 5097 (20.1%) | 5453 (20.3%) | 3786 (15.2%) |
| **Surgical** | 122674 (41.3%) | 13187 (45.6%) | 12337 (43.9%) | 11639 (42.5%) | 14136 (42.9%) | 14590 (43.5%) | 13682 (41.9%) | 15829 (43.2%) | 8586 (33.8%) | 9080 (33.8%) | 9608 (38.6%) |
| **Emergency** | 101383 (34.1%) | 9233 (31.9%) | 9305 (33.1%) | 9285 (33.9%) | 11308 (34.3%) | 11295 (33.6%) | 11315 (34.7%) | 12534 (34.2%) | 8737 (34.4%) | 9134 (34.0%) | 9237 (37.2%) |
| **Other ICU** | 18624 (6.3%) | 1578 (5.5%) | 1739 (6.2%) | 1735 (6.3%) | 1860 (5.6%) | 1988 (5.9%) | 2112 (6.5%) | 2228 (6.1%) | 1973 (7.8%) | 1848 (6.9%) | 1563 (6.3%) |
| **High dependency unit** | 8873 (3.0%) | 751 (2.6%) | 676 (2.4%) | 785 (2.9%) | 923 (2.8%) | 917 (2.7%) | 854 (2.6%) | 938 (2.6%) | 987 (3.9%) | 1376 (5.1%) | 666 (2.7%) |

ICU: intensive care unit; LOS: length of stay; SD: standard deviation

**Supplementary Table 2.** Details on hospital-acquired infections diagnosed in intensive care unit during the study period

|  | **Total** | **2013** | **2014** | **2015** | **2016** | **2017** | **2018** | **2019** | **2020** | **2021** | **2022** |
| --- | --- | --- | --- | --- | --- | --- | --- | --- | --- | --- | --- |
| **Patients with ICU-HAIs** | 20570 | 1818 | 1865 | 1676 | 1865 | 1979 | 1856 | 2050 | 1319 | 1600 | 1650 |
| **HAIs** | 25964 | 2471 | 2412 | 2303 | 2470 | 2693 | 2682 | 2239 | 2889 | 3527 | 2280 |
| **Mean ICU-HAI per patient** | 1.5 | 1.36 | 1.29 | 1.37 | 1.32 | 1.36 | 1.45 | 1.09 | 2.19 | 2.21 | 1.38 |
| **GNB infections**  **(% on ICU-HAIs)** | 12060 (46.5%) | 1236 | 1199 | 1187 | 1200 | 1264 | 1304 | 969 | 1282 | 1461 | 958 |
| **CR-GNB infections**  **(% on ICU-HAIs)** | 2927 (11.3%) | 337 | 303 | 321 | 320 | 305 | 262 | 222 | 312 | 328 | 217 |
| **PA infections**  **(% on ICU-HAIs)** | 4818  (18.6%) | 439 (17.77%) | 448 (18.57%) | 461 (20.02%) | 445 (18.02%) | 547 (20.31%) | 540 (20.13%) | 413 (18.45%) | 513 (17.8%) | 593 (16.8%) | 419 (18.4%) |
| **CRPA infections**  **(% on PA infections)** | 1049  (21.8%) | 114  (26 %) | 90  (20.1 %) | 109  (23.6 %) | 110  (24.7 %) | 109  (19.9 %) | 84  (15.6 %) | 81  (19.6 %) | 106 (20.7 %) | 140 (23.6 %) | 106 (25.3 %) |
| ***Acinetobacter* spp. infections**  **(% on ICU-HAIs)** | 2183  (8.4%) | 303 (12.26%) | 265 (10.99%) | 232 (10.07%) | 243 (9.84%) | 214 (7.95%) | 202 (7.53%) | 124 (5.54%) | 236 (8.2%) | 244 (6.9%) | 120 (5.3%) |
| **CR-*Acinetobacter* spp infections**  **(% on *Acinetobacter* spp infections)** | 1893  (86.7%) | 262 (86.5%) | 239 (90.2%) | 206 (88.8%) | 221 (90.6%) | 181 (84.6%) | 163 (80.7%) | 98 (79.0%) | 217 (91.6%) | 209 (86.7%) | 97 (80.8%) |
| ***Klebsiella* spp. infections**  **(% on ICU-HAIs)** | 5059  (19.5%) | 494 (19.99%) | 486 (20.15%) | 494 (21.45%) | 512 (20.73 %) | 503 (18.68 %) | 562 (20.95 %) | 432 (19.29 %) | 533 (18.4%) | 624 (17.7%) | 419 (18.4%) |
| **CR-Klebsiella spp. infections**  **(% on *Klebsiella* spp infections)** | 1588  (31.4%) | 182 (36.8 %) | 187 (38.5 %) | 186 (37.7 %) | 188  (36.7 %) | 163  (32.4 %) | 139  (24.7 %) | 115  (26.6 %) | 187 (35.1 %) | 153 (24.5 %) | 88  (21 %) |

ICU: intensive care unit; HAI: hospital-acquired infection; GNB: gram-negative bacteria; CR: carbapenem-resistant; CR-GNB: carbapenem-resistant gram-negative bacteria; PA: *Pseudomonas aeruginosa*; CRPA: carbapenem-resistant *Pseudomonas aeruginosa*

**Supplementary Table 3.** Details on infections by *Pseudomonas aeruginosa* acquired in intensive care unit during the study period

|  | **2013** | **2014** | **2015** | **2016** | **2017** | **2018** | **2019** | **2020** | **2021** | **2022** |
| --- | --- | --- | --- | --- | --- | --- | --- | --- | --- | --- |
| **Total VAP episodes** | 856 | 756 | 699 | 798 | 763 | 827 | 714 | 1237 | 1664 | 946 |
| **VAP by PA**  **(% on VAP episodes)** | 183  (21.38 %) | 170  (22.49 %) | 155  (22.17 %) | 188  (23.56 %) | 187  (24.51 %) | 207  (25.03 %) | 174  (24.37 %) | 250 (20.2%) | 333  (20%) | 208  (22%) |
| **VAP by CRPA**  **(% on VAP by PA)** | 60  (32.8 %) | 32  (18.8 %) | 45  (29 %) | 43  (22.9 %) | 41  (21.9 %) | 35  (16.9 %) | 35  (20.1 %) | 56  (22.4 %) | 82  (24.6 %) | 58  (27.9 %) |
| **Total BSI episodes** | 283 | 257 | 256 | 258 | 308 | 315 | 285 | 348 | 392 | 238 |
| **BSI by PA**  **(% on BSI episodes)** | 20  (7.07 %) | 21  (8.17 %) | 22  (8.59 %) | 20  (7.75 %) | 27  (8.77 %) | 30  (9.52 %) | 24  (8.42 %) | 25  (7.2%) | 24  (6.1%) | 10  (4.2%) |
| **BSI by CRPA**  **(% on BSI by PA)** | 3  (15 %) | 7  (33.3 %) | 4  (18.2 %) | 9  (45 %) | 4  (14.8 %) | 4  (13.3 %) | 2  (8.3 %) | 6  (24 %) | 7  (29.2 %) | 3  (30 %) |
| **Total UTI episodes** | 106 | 94 | 83 | 89 | 86 | 71 | 67 | 431 | 567 | 365 |
| **UTI by PA**  **(% on UTI episodes)** | 12  (11.32 %) | 12  (12.77 %) | 8  (9.64 %) | 8  (8.99 %) | 16  (18.6 %) | 11  (15.49 %) | 10  (14.93 %) | 65 (15.1%) | 74 (13.1%) | 53 (14.5%) |
| **UTI by CRPA** | 3  (25 %) | 2  (16.7 %) | 4  (50 %) | 0  (0 %) | 1  (6.2 %) | 1  (9.1 %) | 1  (10 %) | 12  (18.5 %) | 13  (17.6 %) | 10  (18.9 %) |
| **Total IAI episodes** | 219 | 196 | 216 | 199 | 230 | 250 | 186 | 84 | 164 | 177 |
| **IAI by PA**  **(% on IAI episodes)** | 33  (15.07 %) | 36  (18.37 %) | 33  (15.28 %) | 38  (19.1 %) | 47  (20.43 %) | 50  (20 %) | 40  (21.51 %) | 19  (22.6%) | 28  (17.1%) | 37  (20.9%) |
| **IAI by CRPA**  **(% on IAI by PA)** | 12  (36.4 %) | 7  (19.4 %) | 11  (33.3 %) | 16  (42.1 %) | 15  (31.9 %) | 12  (24 %) | 13  (32.5 %) | 4  (21.1 %) | 8  (28.6 %) | 11  (29.7 %) |

VAP: ventilator associated-pneumonia; PA: *Pseudomonas aeruginosa;* CRPA: carbapenem-resistant *Pseudomonas aeruginosa;* BSI: bloodstream infection; UTI: urinary tract infection; IAI: intrabdominal infection

**Supplementary Table 4.** Details on infections by *Klebsiella* spp. acquired in intensive care unit during the study period

|  | **2013** | **2014** | **2015** | **2016** | **2017** | **2018** | **2019** | **2020** | **2021** | **2022** |
| --- | --- | --- | --- | --- | --- | --- | --- | --- | --- | --- |
| **Total VAP episodes** | 856 | 756 | 699 | 798 | 763 | 827 | 714 | 1237 | 1664 | 946 |
| **VAP by *Klebsiella* spp.**  **(% on VAP episodes)** | 211  (24.65 %) | 179  (23.68 %) | 188  (26.9 %) | 210  (26.32 %) | 160  (20.97 %) | 227  (27.45 %) | 182  (25.49 %) | 250 (20.2%) | 348 (20.9%) | 194 (20.5%) |
| **VAP by CR-*Klebsiella***  **(% on VAP by *Klebsiella*)** | 74  (35.1 %) | 72  (40.2 %) | 75  (39.9 %) | 72  (34.3 %) | 52  (32.5 %) | 57  (25.1 %) | 49  (26.9 %) | 101  (40.4 %) | 89  (25.6 %) | 46  (23.7 %) |
| **Total BSI episodes** | 283 | 257 | 256 | 258 | 308 | 315 | 285 | 348 | 392 | 238 |
| **BSI by *Klebsiella* spp.**  **(% on BSI episodes)** | 34  (12.01 %) | 40  (15.56 %) | 44  (17.19 %) | 52  (20.16 %) | 52  (16.88 %) | 46  (14.6 %) | 50  (17.54 %) | 63 (18.1%) | 54 (13.8%) | 32 (13.4%) |
| **BSI by CR-*Klebsiella***  **(% on BSI by *Klebsiella*)** | 10  (29.4 %) | 19  (47.5 %) | 12  (27.3 %) | 22  (42.3 %) | 22  (42.3 %) | 13  (28.3 %) | 17  (34 %) | 27  (42.9 %) | 13  (24.1 %) | 6  (18.8 %) |
| **Total UTI episodes** | 106 | 94 | 83 | 89 | 86 | 71 | 67 | 431 | 567 | 365 |
| **UTI by *Klebsiella* spp.**  **(% on UTI episodes)** | 23  (21.7 %) | 10  (10.64 %) | 22  (26.51 %) | 15  (16.85 %) | 21  (24.42 %) | 16  (22.54 %) | 10  (14.93 %) | 58  (13.5%) | 71  (12.5%) | 51  (14%) |
| **UTI by CR-*Klebsiella*** | 13  (56.5 %) | 3  (30 %) | 13  (59.1 %) | 6  (40 %) | 10  (47.6 %) | 5  (31.2 %) | 4  (40 %) | 17  (29.3 %) | 24  (33.8 %) | 14  (27.5 %) |
| **Total IAI episodes** | 219 | 196 | 216 | 199 | 230 | 250 | 186 | 84 | 164 | 177 |
| **IAI by *Klebsiella***  **(% on IAI episodes)** | 44  (20.09 %) | 44  (22.45 %) | 51  (23.61 %) | 47  (23.62 %) | 42  (18.26 %) | 50  (20 %) | 30  (16.13 %) | 19  (22.6%) | 29  (17.7%) | 32  (18.1%) |
| **IAI by CR-*Klebsiella***  **(% on IAI by *Klebsiella*)** | 28  (63.6 %) | 23  (52.3 %) | 21  (41.2 %) | 25  (53.2 %) | 18  (42.9 %) | 22  (44 %) | 11  (36.7 %) | 6  (31.6 %) | 14  (48.3 %) | 12  (37.5 %) |

VAP: ventilator associated-pneumonia; CR: carbapenem-resistant*;* BSI: bloodstream infection; UTI: urinary tract infection; IAI: intrabdominal infection

**Supplementary Table 5.** Details on infections by *Acinetobacter* spp. acquired in intensive care unit during the study period

|  | **2013** | **2014** | **2015** | **2016** | **2017** | **2018** | **2019** | **2020** | **2021** | **2022** |
| --- | --- | --- | --- | --- | --- | --- | --- | --- | --- | --- |
| **Total VAP episodes** | 856 | 756 | 699 | 798 | 763 | 827 | 714 | 1237 | 1664 | 946 |
| **VAP by *Acinetobacter* spp.**  **(% on VAP episodes)** | 167 (19.51 %) | 120 (15.87 %) | 113 (16.17 %) | 132 (16.54 %) | 104 (13.63 %) | 98  (11.85 %) | 59  (8.26 %) | 171  (13.8%) | 130  (7.8%) | 52 (5.5%) |
| **VAP by CR-** ***Acinetobacter***  **(% on VAP by *Klebsiella*)** | 146  (87.4 %) | 108  (90.0 %) | 103  (91.1 %) | 124  (93.9 %) | 90  (86.5%) | 81  (82.6 %) | 50  (84.7%) | 160  (93.6 %) | 113  (86.9 %) | 41  (78.8 %) |
| **Total BSI episodes** | 283 | 257 | 256 | 258 | 308 | 315 | 285 | 348 | 392 | 238 |
| **BSI by *Acinetobacter* spp.**  **(% on BSI episodes)** | 14  (4.95 %) | 14  (5.45 %) | 12  (4.69 %) | 11  (4.26 %) | 14  (4.55 %) | 12  (3.81 %) | 15  (5.26 %) | 16  (4.6%) | 27  (6.9%) | 12  (5%) |
| **BSI by CR-** ***Acinetobacter***  **(% on BSI by *Acinetobacter*)** | 12  (85.7 %) | 14  (100.0 %) | 12  (100.0 %) | 10  (90.9 %) | 11  (78.6 %) | 9  (75.0 %) | 12  (80.0 %) | 16  (100.0 %) | 22  (81.5 %) | 10  (83.3 %) |
| **Total UTI episodes** | 106 | 94 | 83 | 89 | 86 | 71 | 67 | 431 | 567 | 365 |
| **UTI by *Acinetobacter* spp.**  **(% on UTI episodes)** | 16  (15.09 %) | 8  (8.51 %) | 7  (8.43 %) | 7  (7.87 %) | 5  (5.81 %) | 2  (2.82 %) | 2  (2.99 %) | 10  (2.3%) | 13  (2.3%) | 7  (1.9%) |
| **UTI by CR-** ***Acinetobacter*** | 13  (81.2 %) | 8  (100.0 %) | 7  (100.0 %) | 7  (100.0 %) | 5  (100.0 %) | 2  (100.0 %) | 2  (100.0 %) | 8  (80.0 %) | 11  (84.6 %) | 7  (100.0 %) |
| **Total IAI episodes** | 219 | 196 | 216 | 199 | 230 | 250 | 186 | 84 | 164 | 177 |
| **IAI by *Acinetobacter***  **(% on IAI episodes)** | 35  (15.98 %) | 23  (11.73 %) | 20  (9.26 %) | 17  (8.54 %) | 14  (6.09 %) | 21  (8.4 %) | 11  (5.91 %) | 7  (8.3%) | 11  (6.7%) | 12  (6.8%) |
| **IAI by CR-** ***Acinetobacter***  **(% on IAI by *Acinetobacter*)** | 34  (97.1 %) | 21  (91.3 %) | 16  (80.0 %) | 16  (94.1 %) | 13  (92.9 %) | 17  (80.9 %) | 11  (100.0 %) | 7  (100.0 %) | 10  (90.9 %) | 12  (100.0 %) |

VAP: ventilator associated-pneumonia; CR: carbapenem-resistant*;* BSI: bloodstream infection; UTI: urinary tract infection; IAI: intrabdominal infection

**Supplementary Figure 1.** *Pseudomonas aeruginosa* carbapenem-resistance trend over years in different infection site


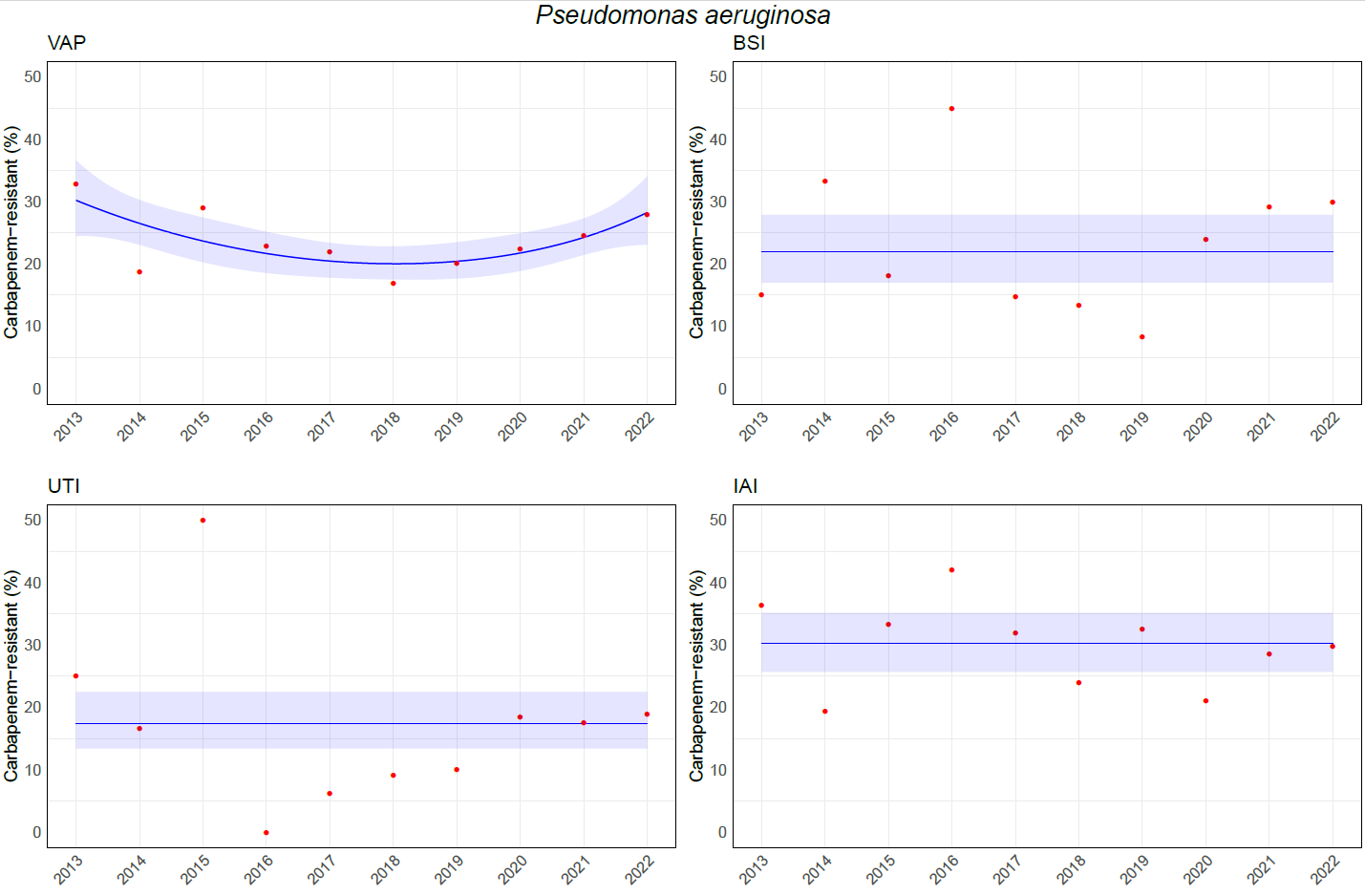


**Supplementary Figure 2.** *Klebsiella* spp. carbapenem-resistance trend over years in different infection site


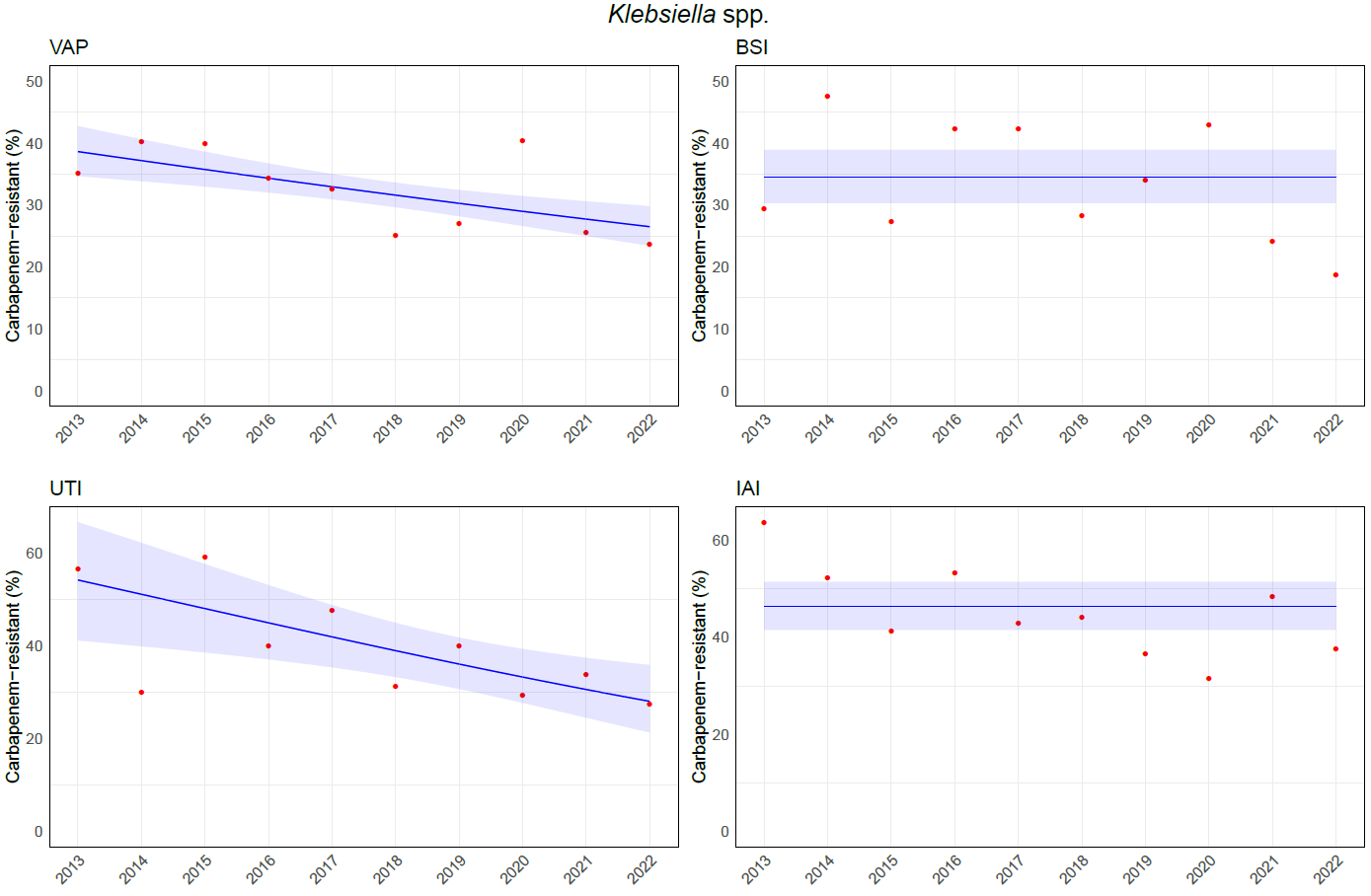


**Supplementary Figure 3.** *Acinetobacter* spp. carbapenem-resistance trend over years in different infection site

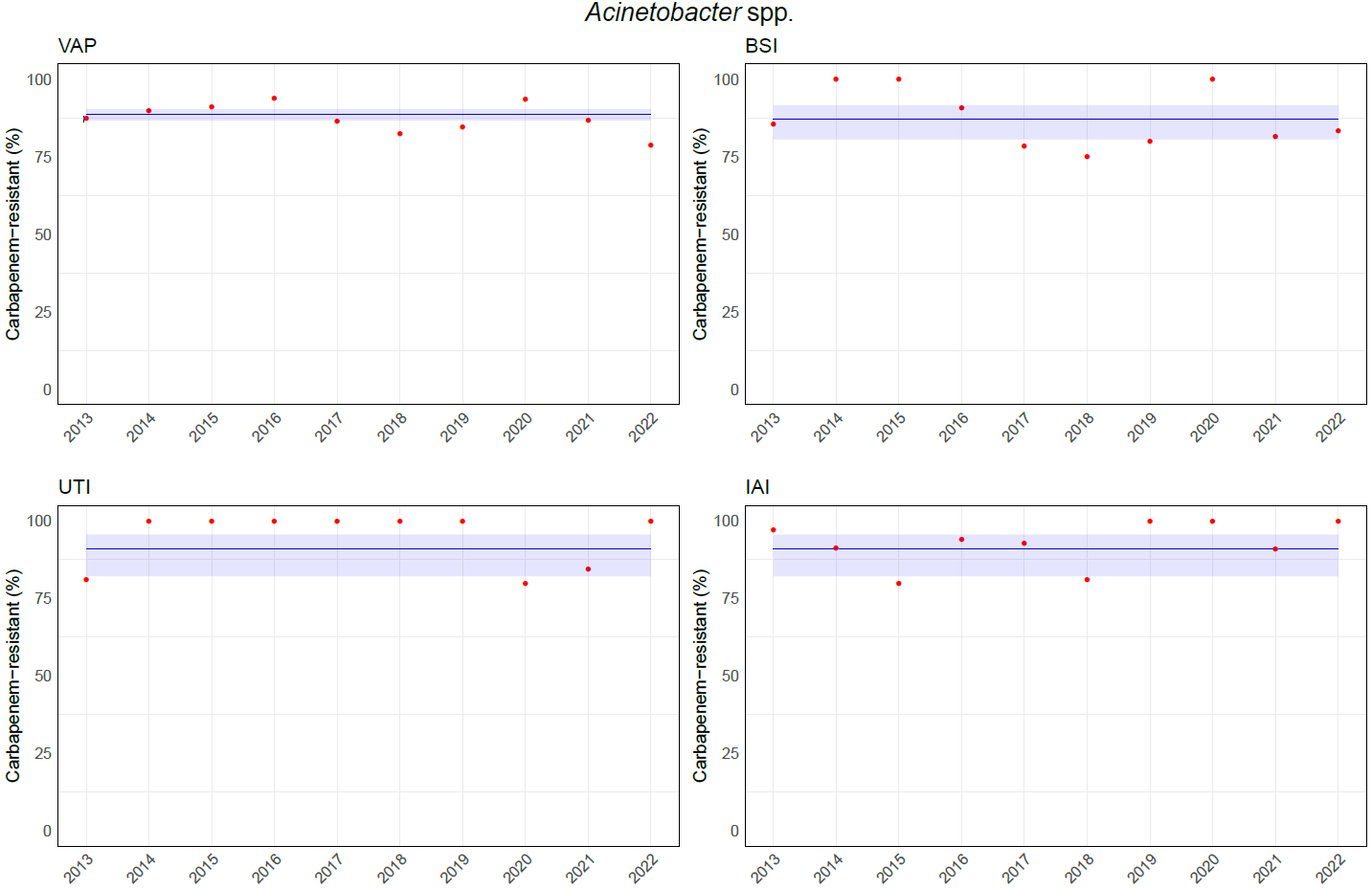


**Supplemental models result.**

**a) Percentage of infections.**

For VAPs we selected the 5^th^ grade orthogonal polynomial:

|  | Estimate | Std. Error | Wald test p-value |
| --- | --- | --- | --- |
| Intercept | –0.622 | 0.013 | < 0.001 |
| 1st degree polynomial | 0.554 | 0.041 | < 0.001 |
| 2nd degree polynomial | 0.419 | 0.042 | < 0.001 |
| 3rd degree polynomial | –0.189 | 0.042 | < 0.001 |
| 4th degree polynomial | –0.272 | 0.041 | < 0.001 |
| 5th degree polynomial | –0.222 | 0.041 | < 0.001 |

which corresponds to:

η = – 0.852 + 0.083*year + 0.083*year^2^ + 0.011*year^3^ – 0.004 *year^4^ – 0.0008*year^5^

where η is the linear predictor and the variable *year* represents the year shifted by the weighted mean of the observations. We will use this notation throughout all the **Supplemental models result**.

The p–value of the log–likelihood ratio test with respect to the null model is: p <0.001.

For BSI there was no significant trend (p–value = 0.615), and the model is:

|  | Estimate | Std. Error | Wald test p-value |
| --- | --- | --- | --- |
| Intercept | –2.058 | 0.019 | < 0.001 |

which corresponds to:

η = – 2.058

For UTI the 4^th^ grade polynomial was chosen through a forward selection (p-value < 0.001):

|  | Estimate | Std. Error | Wald test p-value |
| --- | --- | --- | --- |
| Intercept | –2.808 | 0.030 | < 0.001 |
| 1st degree polynomial | 1.788 | 0.082 | < 0.001 |
| 2nd degree polynomial | 1.211 | 0.093 | < 0.001 |
| 3rd degree polynomial | –0.305 | 0.089 | < 0.001 |
| 4th degree polynomial | –0.77842 | 0.081 | < 0.001 |

which corresponds to the model for the linear predictor:

η = –3.354 + 0.447*year + 0.160*year^2^ – 0.018*year^3^ – 0.006*year^4^

The percentage of IAI showed a linear trend (p–value < 0.001) where the coefficients of the model are:

|  | Estimate | Std. Error | Wald test p-value |
| --- | --- | --- | --- |
| Intercept | –2.563 | 0.024 | < 0.001 |
| 1st degree polynomial | –0.065 | 0.008 | < 0.001 |

which corresponds to:

η = –2.563 – 0.065*year

**b) VAP and CR–BSI incidence rate**

For VAP incidence rate a 5^th^ grade polynomial was selected (p–value < 0.001). The coefficients of the model are:

|  | Estimate | Std. Error | Wald test p-value |
| --- | --- | --- | --- |
| Intercept | – 4.621 | 0.021 | < 0.001 |
| 1st degree polynomial | 0.531 | 0.018 | < 0.01 |
| 2nd degree polynomial | 0.388 | 0.005 | < 0.001 |
| 3rd degree polynomial | – 0.122 | 0.003 | < 0.001 |
| 4th degree polynomial | – 0.150 | 0.0002 | < 0.001 |
| 5th degree polynomial | – 0.09 | 0.0001 | < 0.01 |

that corresponds to:

η = – 4.812 + 0.054*year + 0.042*year^2^ + 0.006*year^3^ – 0.001*year^4^ – 0.0003*year^5^

For CR–BSI we stopped at the 4^th^ degree (p–value < 0.001) and the model coefficients are:

|  | Estimate | Std. Error | Wald test p-value |
| --- | --- | --- | --- |
| Intercept | – 6.061 | 0.033 | < 0.001 |
| 1st degree polynomial | 0.447 | 0.015 | < 0.001 |
| 2nd degree polynomial | 0.074 | 0.009 | 0.180 |
| 3rd degree polynomial | – 0.192 | 0.0009 | < 0.001 |
| 4th degree polynomial | – 0.237 | 0.0004 | < 0.001 |

that corresponds to:

η = – 6.1659191 + 0.108*year + 0.040*year^2^ – 0.004*year^3^ – 0.002*year^4^

**c) Multi–drug resistance microorganism**

A U–shaped correlation was found for *Pseudomonas aeruginosa* (p–value < 0.01). The coefficients of the model are:

|  | Estimate | Std. Error | Wald test p-value |
| --- | --- | --- | --- |
| Intercept | –1.278 | 0.035 | < 0.001 |
| 1st degree polynomial | –0.046 | 0.109 | 0.67 |
| 2nd degree polynomial | 0.397 | 0.111 | < 0.001 |

which is represented by:

η = –1.419 – 0.012*year + 0.017*year^2^

For *Klebsiella* spp. a linear trend was found (p-value < 0.001), where the coefficients of the model are:

|  | Estimate | Std. Error | Wald test p-value |
| --- | --- | --- | --- |
| Intercept | –0.789 | 0.030 | <0.001 |
| 1st degree polynomial | –0.736 | 0.098 | <0.001 |

that corresponds to:

η = – 0.830 – 0.081*year

*Acinetobacter* spp. did not show any trend (p-value = 0.065). The corresponding null model is:

|  | Estimate | Std. Error | Wald test p-value |
| --- | --- | --- | --- |
| Intercept | 1.876 | 0.063 | <0.001 |

that is:

η = 1.876

**d) Carbapenem–resistant microorganisms**

***Pseudomonas aeruginosa***

We analysed the presence of carbapenem–resistant *Pseudomonas aeruginosa* according to the different infection site. The only infections where a trend for MDR was found are VAPs, which have a U–shaped relation for the linear predictor (p–value < 0.01) and the model coefficients are:

|  | Estimate | Std. Error | Wald test p-value |
| --- | --- | --- | --- |
| Intercept | –1.177 | 0.053 | < 0.001 |
| 1st degree polynomial | –0.135 | 0.157 | 0.391 |
| 2nd degree polynomial | 0.559 | 0.165 | < 0.001 |

corresponding to the following relation:

η = –1.38 – 0.009*year + 0.024*year^2^

For BSI there is no trend (p-value = 0.941) and the estimate is:

|  | Estimate | Std. Error | Wald test p-value |
| --- | --- | --- | --- |
| Intercept | –1.267 | 0.162 | <0.001 |

that corresponds to:

η = –1.267

Also for UTI there is no trend (p-value = 0.821), and the estimate is:

|  | Estimate | Std. Error | Wald test p-value |
| --- | --- | --- | --- |
| Intercept | –1.552 | 0.161 | <0.001 |

that corresponds to:

η = –1.552

There is no trend for IAI either (p-value = 0.905) with coefficient:

|  | Estimate | Std. Error | Wald test p-value |
| --- | --- | --- | --- |
| Intercept | –0.838 | 0.115 | <0.001 |

that corresponds to:

η = –0.838

***Klebsiella* spp.**

*Klebsiella* spp. showed a tendency to decrease with a linear trend in VAP (p–value < 0.001). The coefficients of the models are:

|  | Estimate | Std. Error | Wald test p-value |
| --- | --- | --- | --- |
| Intercept | –0.742 | 0.046 | <0.001 |
| 1st degree polynomial | –0.562 | 0.145 | <0.001 |

which corresponds to:

η = – 0.773 – 0.062*year

We notice a decreasing trend also for UTI (p–value < 0.01), which model reads:

|  | Estimate | Std. Error | Wald test p-value |
| --- | --- | --- | --- |
| Intercept | –0.385 | 0.132 | <0.01 |
| 1st degree polynomial | –1.119 | 0.379 | <0.01 |

which corresponds to:

η = – 0.447 – 0.123*year

For BSI (p-value = 0.133) there was no trend, where respectively:

|  | Estimate | Std. Error | Wald test p-value |
| --- | --- | --- | --- |
| Intercept | –0.642 | 0.097 | <0.001 |

i.e.

η = – 0.642

There is no trend for IAI either (p-value = 0.905), with coefficient:

|  | Estimate | Std. Error | Wald test p-value |
| --- | --- | --- | --- |
| Intercept | –0.145 | 0.102 | 0.156 |

i.e.

η = – 0.145

***Acinetobacter* spp.**

For *Acinetobacter* spp. there was no trend that correlate the presence of carbapenem–resistant strains with time. In particular:

For VAP (p-value = 0.350), the coefficient is:

|  | Estimate | Std. Error | Wald test p-value |
| --- | --- | --- | --- |
| Intercept | 2.056 | 0.093 | <0.001 |

that is:

η = 2.056

For BSI (p-value = 0.237), the coefficient is:

|  | Estimate | Std. Error | Wald test p-value |
| --- | --- | --- | --- |
| Intercept | 1.908 | 0.2458 | <0.001 |

that is:

η = 1.908

For UTI (p-value = 0.931), the coefficient is:

|  | Estimate | Std. Error | Wald test p-value |
| --- | --- | --- | --- |
| Intercept | 2.303 | 0.396 | <0.001 |

that is:

η = – 2.303

For IAI (p-value = 0.350), the coefficient is:

|  | Estimate | Std. Error | Wald test p-value |
| --- | --- | --- | --- |
| Intercept | 2.417 | 0.279 | <0.001 |

that is:

η = – 2.417
